# The role of mitochondria in the peri‐implant microenvironment

**DOI:** 10.1113/EP090988

**Published:** 2023-01-17

**Authors:** Xidan Zhang, Jiyu Sun, Min Zhou, Chen Li, Zhuoli Zhu, Xueqi Gan

**Affiliations:** ^1^ State Key Laboratory of Oral Diseases National Clinical Research Center for Oral Diseases West China Hospital of Stomatology Sichuan University Chengdu China

**Keywords:** mitochondria, osseointegration, peri‐implant

## Abstract

Osseointegration is a dynamic biological process in the local microenvironment adjacent to a bone implant, which is crucial for implant performance and success of the implant surgery. Recently, the role of mitochondria in the peri‐implant microenvironment during osseointegration has gained much attention. Mitochondrial regulation has been verified to be essential for cellular events in osseointegration and as a therapeutic target for peri‐implant diseases in the peri‐implant microenvironment. In this review, we summarize our current knowledge of the key role of mitochondria in the peri‐implant milieu, including the regulation of mitochondrial reactive oxygen species and mitochondrial metabolism in angiogenesis, the polarization of macrophage immune responses, and bone formation and resorption during osseointegration, which will contribute to the research field and the development of new treatment strategies to improve implant success. In addition, we indicate limitations in our current understanding of the regulation of mitochondria in osseointegration and suggest topics for further study.

## Abstract (highlights)

- **What is the topic of this review?**
  In this review, we consider the key role of mitochondria in the peri‐implant milieu, including the regulation of mitochondrial reactive oxygen species and mitochondrial metabolism in angiogenesis, the polarization of macrophage immune responses, and bone formation and bone resorption during osseointegration.
- **What advances does it highlight?**
  Mitochondria contribute to the behaviours of peri‐implant cell lines based on metabolic and reactive oxygen species signalling modulations, which will contribute to the research field and the development of new treatment strategies for improving implant success.

## 1 INTRODUCTION

**FIGURE 1:**
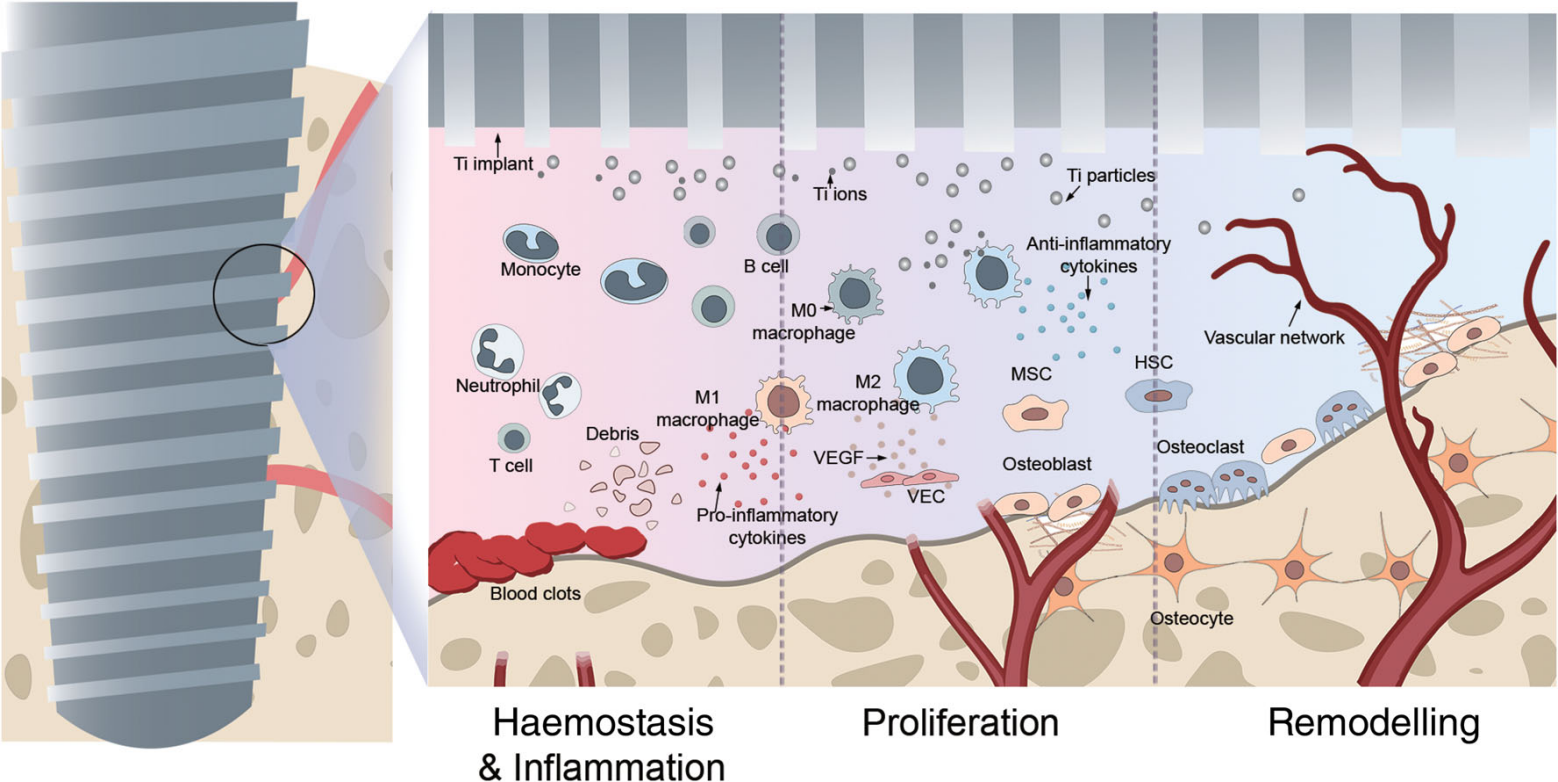
The peri‐implant microenvironment is a complex structural and biological system that contains multiple cells, cytokines and bone marrow. In response to different external stimuli, cellular signalling transduction pathways are affected. The microenvironment includes multilineage haematopoietic and mesenchymal cells, resident bone cells (osteoclasts, osteoblasts and osteocytes), immunological cells (macrophages, T cells, B cells and neutrophils) and endothelial cells. Implant osseointegration is a dynamic biological process that includes haemostasis and inflammation, proliferation and remodelling, and these cells appear chronologically during osseointegration in the peri‐implant microenvironment.

Nowadays, titanium (Ti) implants are used extensively in the fields of orthopaedics and dentistry for spinal and craniofacial reconstructions, joint arthroplasties and dental prostheses. Research in this area is crucial owing to the large‐scale use of these screws in medical practice. Titanium implant osseointegration is a biological process that involves immuno‐inflammatory reactions, angiogenesis, bone formation and bone resorption, all of which are influenced by the peri‐implant microenvironment (Chen et al., 2021). Therefore, the Ti implant–bone interface micro‐milieu is the key factor for implant success. In the process of osseointegration, the peri‐implant microenvironment can be considered as a complex structural and biological system that contains cells, cytokines, bone marrow, bone matrix and metal particles/ions released from the implants. Cells are central elements in the microenvironment, including multi‐lineage haematopoietic and mesenchymal stem cells (MSCs), resident bone cells (osteoclasts, osteoblasts and osteocytes), immune cells (monocytes, macrophages, T cells, B cells and neutrophils) and vascular endothelial cells (VECs) (Zheng et al., 2013), which provide a stable growth environment through well‐orchestrated cellular interplay to maintain implant stability (Figure 1).

Mitochondria are well known as the major energy source for most cells. Carbohydrates, amino acids and lipids are imported into the cell and converted to pyruvate, fatty acids and amino acids, which are oxidized in the tricarboxylic acid (TCA) cycle in the mitochondrial matrix. Through a process known as oxidative phosphorylation (OXPHOS), ATP is produced from the respiratory chain. Now, mitochondria are no longer viewed only as the energy machinery of the cell but also as a vital source of dynamic microenvironmental signals (Bennett et al., 2022; Brand, 2016, 2020; Shen, Pender et al., 2022). In response to different external and internal stimuli, mitochondria commonly change (increase or decrease) mitochondrial energy metabolism, including OXPHOS and activity of the electron transport chain (ETC) to support ATP generation (Bennett et al., 2022). During this, mitochondrial biogenesis and mitochondrial dynamics can change accordingly (Bennett et al., 2022; Sokolova, 2018). In these processes, mitochondrial reactive oxygen species (mROS) are inevitably produced from the ETC, which can act as signals to regulate various cellular processes (Chakrabarty & Chandel, 2022). There is a large literature on the roles of mROS in cellular physiological functions, including cell proliferation, migration, and differentiation (Blajszczak & Bonini, 2017; Chen, Li, Shi et al., 2022; Wang, Fu et al., 2021; Wang, Yang et al., 2020).

In this review, we explore the recent prospective link between mitochondrial regulation and the peri‐implant microenvironment, aiming to elucidate mitochondrial strategies for improving tissue regeneration and bone healing during implant osseointegration.

## 2 PERI‐IMPLANT CELL FUNCTION AND MITOCHONDRIAL REGULATION

The peri‐implant microenvironment is a dynamic structure that undergoes a coordinated and sequentially organized repair mechanism called osseointegration, which consists of three overlapping phases: haemostasis and inflammation; proliferation; and remodelling. Haemostasis begins with the surgical trauma caused by the implant procedure. When the vasculature is disrupted by the introduction of the implant, platelets immediately aggregate from a blood clot at the surgical site (Sun et al., 2022). After that, inflammation is initiated (Sun et al., 2022), during which immune cells, including monocytes, macrophages, neutrophils and multinucleated giant cells, are activated and recruited to the implant site (Pfeiffenberger et al., 2021; Ripszky Totan et al., 2022; Terheyden et al., 2012). Within 24 h of implant insertion, neutrophils dominate the implant site and are replaced by neutrophils, macrophages and monocytes (Terheyden et al., 2012). A favourable immune response not only removes necrotic tissue, but also provides a cell‐instructive peri‐implant microenvironment via release of cytokines and growth factors, which promote MSC recruitment and proliferation, angiogenesis and collagen matrix deposition (Sun et al., 2022; Wang et al., 2016).

After the inflammatory phase, the proliferative phase is initiated by the formation of new extracellular matrix and by angiogenesis. Angiogenesis is stimulated by vascular endothelial growth factor (VEGF) released from macrophages. In response to VEGF, the VECs are activated to initiate remodelling of the vascular network (Ripszky Totan et al., 2022; Terheyden et al., 2012). Also, other growth factors, such as platelet‐derived growth factor from platelets and fibroblast growth factor (FGF) from macrophages, are angiogenic (Terheyden et al., 2012). Blood vessels provide nutrients and oxygen to organs during bone repair. Mesenchymal stem cells from the bone marrow are recruited into the newly developing blood vessels around the implant and differentiate into osteoblasts (Sun et al., 2022). Osteoblasts are influenced by both growth factors and the implant surface topography, where they begin to form bone extracellular matrix and deposit collagen matrix, which is considered to be primary bone formation (Wang et al., 2016). Woven bone, which is relatively unorganized, grows in the bone–implant gap a few days after implantation (Liu, Rath et al., 2020; Wang et al., 2016).

Removal of the woven bone by osteoclasts is the beginning of the remodelling phase, the last phase of osseointegration. During the remodelling phase, osteoclasts resorb the extracellular matrix to remodel the woven bone to lamellar bone, a new three‐dimensional load‐oriented trabecular network around the implant (Sun et al., 2022; Terheyden et al., 2012). The remodelling phase continues for several years until most woven bone and primary bone are replaced by load‐oriented bone and lamellar bone. Importantly, recent osseointegration studies have focused on several cell types, including macrophages, VECs, MSCs, osteoblasts and osteoclasts (Figure 1).

In short, VECs, MSCs, osteoblasts and osteoclasts appear chronologically during osseointegration in the peri‐implant microenvironment (Terheyden et al., 2012), and all of them play direct and indirect roles in wound healing (Table 1). Given the central roles of mitochondria in sustaining cell survival, proliferation and differentiation, recent studies have demonstrated the mitochondrial regulation in these peri‐implant cells, and we focus specifically on the biological events closely related to osseointegration (Table 1).

**TABLE 1:** Mitochondrial roles in the main types of peri‐implant cells involved in osseointegration.

**Cell type**	**Osteointegration phase**	**Biological events**	**References**	**Mitochondrial regulation**	**References**
Macrophages	Haematoma and inflammatory phase (M1 dominated)	Clear tissue debris Promote inflammation	Amengual‐Peñafiel et al. (2021); He et al. (2022); Oishi & Manabe (2018); Terheyden et al. (2012)	Glycolysis increased and oxidative phosphorylation activity decreased	Liu & Ho (2018)
				Tricarboxylic acid cycle impaired, and citrate and succinate elevated	Wang, Li et al. (2021)
				Generation of mitochondrial reactive oxygen species increased following the oxidation of succinate	Mills et al. (2016b)
	Proliferative phase and remodelling phase (M2 dominated)	Promote angiogenesis by secreting vascular endothelial growth factor Promote osteoinduction by secreting growth factors	He et al. (2022); Shen Fang et al. (2022); Terheyden et al. (2012)	Oxidative phosphorylation increased	Liu & Ho (2018)
				The oxidation of succinate decreased	Lampropoulou et al. (2016)
Vascular endothelial cells	The proliferative phase and remodelling phase	Proliferation and migration to form tubes Vascular network remodelling	Diomede et al. (2020); Hu et al. (2018); Terheyden et al. (2012)	Vascular endothelial growth factor promotes endothelial migration in part by mitochondrially generated reactive oxygen species	Wang et al. (2011)
				Vascular endothelial growth factor and fibroblast growth factor promote glycolysis	Rohlenova et al. (2018)
				Mitochondrial biogenesis increased	Wright et al. (2008)
Mesenchymal stem cells	Haematoma and inflammation	Recruited from peripheral circulation; they migrate to the implantation sites and adhere to the surface of implants Differentiate into osteoblasts	Diomede et al. (2020); Palmquist et al. (2010); Terheyden et al. (2012)	Oxidative phosphorylation increased	Shum et al. (2016); Wan et al. (2021)
				Tricarboxylic acid cycle enhanced	Chen, Li, Wei et al. (2022)
				Mitochondrial membrane potential increased	Shum et al. (2016); Wan et al. (2021)
Osteoblasts	The proliferative phase and remodelling phase	Adhesion and proliferation at the implant site Mineralization Differentiation into osteocytes	Bahney et al. (2019); Hadjidakis & Androulakis (2006); Terheyden et al. (2012)	Mitochondrial biogenesis increased ATP production increased Mitochondrial membrane potential increased	Fukai & Ushio‐Fukai (2020)
Osteoclasts	Remodelling phase	Recruited from blood monocytes and migrate to the implantation site Remove the damaged bone	Hadjidakis & Androulakis (2006); He et al. (2022); Terheyden et al. (2012)	Mitochondrial biogenesis increased Oxygen consumption increased Oxidative phosphorylation increased Mitochondrial reactive oxygen species generation increased	Lemma et al. (2016); Zheng et al. (2020)

In the very early stages of the inflammatory phase, the immune system is activated and is dominated by macrophages, which are important components of the bone marrow (Terheyden et al., 2012). Macrophages are classically divided into M1 macrophages (pro‐inflammatory type) and M2 macrophages (anti‐inflammatory type). The M1 macrophages initiate the necessary inflammatory responses and induce osteoclastogenesis by secreting inflammatory cytokines (interleukin‐6, interleukin‐1β and tumor necrosis factor‐α) during the early inflammatory phase (Amengual‐Peñafiel et al., 2021; Kubatzky et al., 2018; Oishi & Manabe, 2018); however, extensive M1 infiltration can cause peri‐implantitis (Wang, Li et al., 2020). In contrast, the M2 macrophages are responsible for wound healing by anti‐inflammation, angiogenesis and osteoinduction in the proliferative phase (Hu & Olsen, 2016; Jetten et al., 2014; Shen Fang et al., 2022). An efficient and timely transformation from the M1 to M2 macrophage phenotype can terminate the inflammatory response and release VEGF and osteogenic cytokines, which are crucial for bone repair (Amengual‐Peñafiel et al., 2021; Wang, Li et al., 2020). Therefore, it is the macrophage polarization states and not a specific phenotype that determines integration of implants. Mitochondria play a key role in macrophage activation and cytokine production, which, in turn, promotes the macrophage phenotypic change (Wang, Li et al., 2021). Mitochondrial ROS are considered to be an essential part of the antibacterial response and inflammatory cytokine production (Mills & O'Neill, 2016). Moreover, mitochondrial metabolism and metabolites such as citrate, succinate and itaconate can influence macrophage activation (Mills & O'Neill, 2016; Wang, Li et al., 2021). Manipulating the mitochondrial metabolism and mROS generation by macrophages could reprogram macrophages and thus reshape the peri‐implant microenvironment to a favourable state (He et al., 2021; Qing et al., 2020; Yuan et al., 2019).

Vascular endothelial cells are the inner lining of the bone vascular system, which provides nutrients and metabolites necessary for osteogenesis (including the migration, proliferation and differentiation of osteogenesis‐related cells), aiding in bone regeneration (Zhou et al., 2020). After the mechanical damage caused by the implantation procedure, there will be temporary hypoxia in the peri‐implant region owing to vascular disruption and oxygen consumption by metabolically active cells (Kumar et al., 2021; Vaidya et al., 2017; Zou et al., 2011). During hypoxia, macrophages secret VEGF, which promotes the proliferation, migration and angiogenesis of VECs (Guo et al., 2017). Mitochondria are considered to be the central oxygen sensors in the vasculature (Davidson & Duchen, 2007). In response to hypoxia, production of mROS increases and the mROS escape into the cytoplasm (Zhang & Gutterman, 2007). Wang et al. (2011) revealed that mROS regulate migration of VECs via Rac1 activation. Moreover, several studies have demonstrated the mitochondrial functions are promoted in response to VEGF in VECs during angiogenesis, including mitochondrial oxidative respiration and mitochondrial biogenesis (Guo et al., 2017; Wright et al., 2008). These findings reveal a possible important contribution of mitochondrial regulation in angiogenesis during wound healing after implant surgery.

Mesenchymal stem cells are crucial components in the peri‐implant microenvironment. After angiogenesis, MSCs are recruited from the peripheral circulation. They migrate to the implantation site and adhere to the surface of the implant, and their proliferation and differentiation into bone‐forming osteoblasts are crucial for successful osseointegration (Palmquist et al., 2010). After the implant surface is populated by relatively immature MSCs, the established local environment and systemic factors converge to control osteoinduction and osteogenesis and to influence osseointegration, which involves the mitochondria. During osteogenic differentiation, increased mitochondrial membrane potential, intracellular ATP content and mROS production are present together with OXPHOS (Shum et al., 2016; Wan et al., 2021). Consistent with this, Chen, Li, Wei et al. (2022) proved that boosting the TCA cycle of MSCs by increasing the mitochondrial membrane potential and glucose uptake can enhance osteogenic differentiation and new bone formation on the titanium implant surface, highlighting the important role played by mitochondria in osteogenesis, which is crucial for osseointegration. Moreover, mitochondrial compromise might be a crucial contributor to MSC dysfunction in the peri‐implant microenvironment. Shum et al. (2020) measured the mitochondrial function of bone marrow stem cells obtained from patients offered spinal instrumentation fusion surgery using a potentiometric probe and CMXRos staining (a red‐fluorescent dye that stains mitochondria and indicates the MMP levels) and suggested that patients with a lower CMXRos signal, and thus lower mitochondrial OXPHOS function, had poor osseointegration.

Osteogenic differentiation is the process by which MSCs form osteoblasts. Osteoblasts secrete collagen matrix to initiate new bone formation. Mature osteoblasts are surrounded by a secretory matrix and eventually differentiate into osteocytes, which are the central cells of mineralized bone and can respond to mechanical stress in the peri‐implant microenvironment (Bahney et al., 2019; Hadjidakis & Androulakis, 2006; Terheyden et al., 2012). Consistent with MSCs, during osteoblast differentiation, robust mitochondrial biogenesis was observed, accompanied by increased ATP production, oxygen consumption, antioxidant capacity and decreased mitochondrial stress (Fukai & Ushio‐Fukai, 2020). Moreover, mitochondrial dysfunction is a crucial mechanism that impairs osteoblasts in pathological states, especially in diabetes, and contributes to implant loosening (Hu et al., 2018; Wang, Fu et al., 2021). Hu et al. (2018) demonstrated that diabetes induced mitochondrial dysfunction of osteoblasts, including reduced production of ATP and mitochondrial membrane potential, in addition to damage to the mitochondrial structure, such as a swollen appearance, disarrangement and ablation of the cristae and a lower electron density of the mitochondrial matrix, leading to osteoblast dysfunction and poor bone regeneration in the peri‐implant microenvironment. Moreover, Ti particles induced osteoblast apoptosis via activation of the mitochondrial caspase‐dependent pathways (Bressan et al., 2019; Yang et al., 2019). Notably, mROS, a natural byproduct of oxidative metabolism, are crucial regulators of the cellular function of MSCs and osteoblasts and contribute to numerous pathological conditions in the peri‐implant microenvironment (Borys et al., 2019; Chen, Wang, Li et al., 2022; Ghensi et al., 2017; Mijiritsky et al., 2020), as discussed in the next section. Therefore, normal mitochondrial function and mitochondrial homeostasis are crucial for bone formation in the peri‐implant microenvironment.

In the late stages of osteointegration, bone remodelling is mostly dependent on osteoclasts, which are bone‐resorbing cells derived from myeloid progenitors and osteal macrophages (Terheyden et al., 2012). They are responsible for resorption of mineralized tissue, which can make room for new bone formation and remove primary bone‐implant contacts (Terheyden et al., 2012). During osteoclastogenesis, mitochondrial biogenesis and metabolism are upregulated, oxygen consumption is increased, and OXPHOS predominates for obtaining energy (Lemma et al., 2016; Zheng et al., 2020). This is supported by findings of increased expression of genes related to mitochondrial biogenesis and function during osteoclast differentiation, including peroxisome proliferator‐activated receptor‐gamma coactivator 1β (PGC1β), peroxisome proliferator‐activated receptor γ (PPARγ) and estrogen‐related receptor α (ERRα) (Park‐Min, 2019). Receptor activator of nuclear factor kappa‐Β ligand (RANKL)‐mediated osteoclast development induces PGC1 via ROS‐activated cAMP response element‐binding protein (CREB), and PGC1‐deficient animals have increased bone density and diminished osteoclast function by suppression of mitochondrial biogenesis (Gu et al., 2017). In osteoclasts, activation of PPARγ with rosiglitazone and RANKL increased PGC1β expression and ERRα expression (Park‐Min, 2019; Yang & Wan, 2019). Interestingly, deletion of the ERRα gene completely prevented rosiglitazone‐induced activation of mitochondrial biogenesis in osteoclasts (Yao et al., 2021). Mitochondria also create ROS as a byproduct of ATP production. Therefore, endogenous mROS generation is also induced in osteoclasts in response to stimulation by macrophage colony‐stimulating factor and RANKL (Agidigbi & Kim, 2019). Mitochondrial ROS play an important role in ROS generation and subsequent signalling cascades in osteoclasts. When cytosolic Ca^2+^ is released in response to mROS generation, the TCA cycle in osteoclasts is activated, further speeding up mROS production (Tao, Ge et al., 2020). Moreover, Ti ions released from implants stimulate osteoclasts to increase the production of mROS in the peri‐implant microenvironment (Borys et al., 2019; Zhu et al., 2021). These upregulated mROS can subsequently promote the activity of osteoclasts and thus facilitate osteoclastogenesis (Agidigbi & Kim, 2019; Zhu et al., 2021). However, most of the few studies that have focused on the mitochondrial regulation of osteoclasts in osseointegration were in vitro studies; consequently, the complex connection between mitochondria and osteoclasts in vivo requires further clarification.

In summary, in the peri‐implant microenvironment the mitochondria play a central role in regulation of cellular events that are essential for implant osseointegration. The detailed mechanisms underlying how mROS and metabolism regulate the functions of these cells are discussed in the next section.

## 3 MITOCHONDRIAL REACTIVE OXYGEN SPECIES IN THE PERI‐IMPLANT MICROENVIRONMENT

### 3.1 Sources of mitochondrial ROS in the peri‐implant microenvironment

Mitochondria are considered to be the principal producers of ROS. In the process of OXPHOS, which is coupled with mitochondrial respiration, electron leaks from the ETC at complex I and III, followed by the production of the superoxide anion (•O^2−^) and H_2_O_2_. Most of the free radicals are termed ‘mitochondrial ROS’ in the literature (Brand, 2016).

Reactive oxygen species can arise at all stages of osseointegration. In the initial phase, the wound and tissue damage caused by surgical implantation procedures will release ROS directly (Mouthuy et al., 2016; Zhang et al., 2010). These ROS in the peri‐implant microenvironment will induce further endogenous mROS generation. The implant surgery will create a temporarily hypoxic microenvironment, owing to restriction of the blood supply to tissues. In the ischaemic microenvironment, hypoxia initiates a sequence of metabolic reactions that increase ROS production, especially in mitochondria (Fuhrmann & Brüne, 2017; Reichard & Asosingh, 2019). Moreover, the initial inflammatory response will contribute to ROS production and amplification, which is mainly regulated by mitochondria during osseointegration.

Given that bone repair and bone remodelling are energy‐consuming processes, mROS are inevitably generated during mitochondrial ATP production. However, the mROS‐specific contribution to total ROS production during osseointegration in the peri‐implant microenvironment remains unclear owing to the existence of other sources of ROS. Borys et al. (2019) demonstrated that the activity of mitochondrial complex I was significantly reduced in the periosteum‐like tissue adhering to the titanium implant in comparison to the control group periosteum. This indicated that the source of ROS in the peri‐implant microenvironment might be disturbances in mitochondrial complex I. Moreover, a study with a model of human peri‐implantitis found that NADPH oxidase and NADPH oxidase 4 (*NOX4*), which are mitochondrial ROS production related‐genes, were upregulated in peri‐implantitis (Mijiritsky et al., 2020). In the peri‐implant microenvironment, metal particles released from the implant also contribute to the production of ROS by inducing mitochondrial dysfunction. Titanium particles induce a significant decrease in antioxidant enzymes, including superoxide dismutase 1 (SOD1), superoxide dismutase 2 (SOD2) and transcript levels of sirtuin 1 (*SIRT1*), which results in mitochondrial malfunction and overproduction of mROS (Bressan et al., 2019; Zhang, Zhu et al., 2020).

**FIGURE 2:**
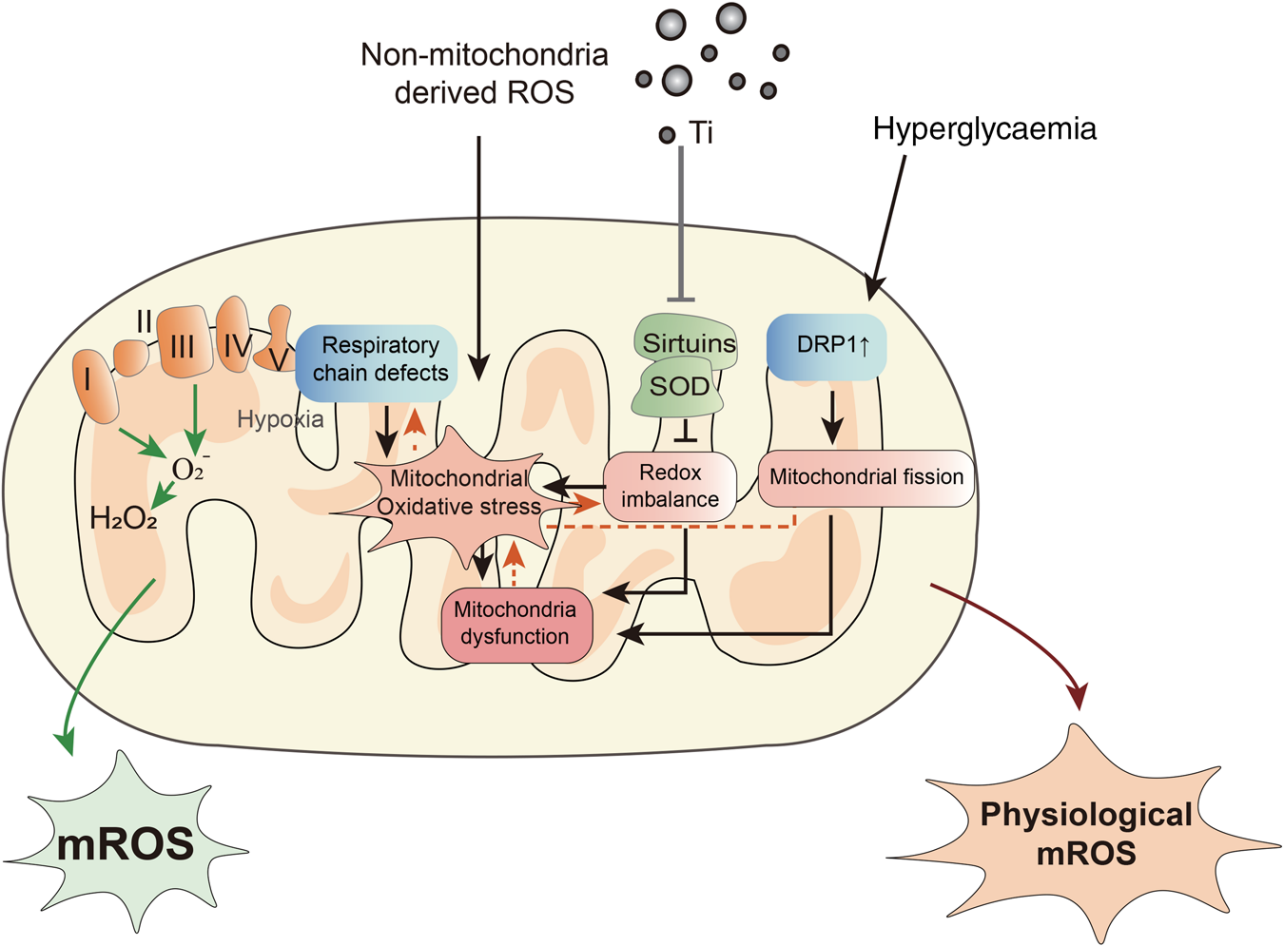
Source of mitochondrial reactive oxygen species (mROS). Mitochondria are an unavoidable source of reactive oxygen species (ROS) from oxidative metabolism during implant osseointegration. The ROS arise from the biological response to implantation and can induce further endogenous ROS generation via the mitochondrial electron transport chain. Moreover, Ti particles released from the implant also contribute to the production of ROS by impairing mitochondrial antioxidant capacity. Hyperglycaemia causes excessive ROS by upregulating DRP1.

In pathological states, mitochondria are not only the main source but also the main target of ROS. Excessive mitochondrial division in diabetics is caused by elevated circulating glucose and free fatty acids, leading to an increase in dynamin‐related protein 1 (DRP1) and an increase in ROS levels in the peri‐implant cells (Caja & Enriquez, 2017; Hu et al., 2017, 2018). Oxidative stress caused by excess ROS can lead to permanent mitochondrial damage, which, in turn, enhances ROS‐induced ROS production (Hu et al., 2018; Zorov et al., 2014). Therefore, mitochondria‐derived ROS can be considered an important part of the peri‐implant microenvironment (Figure 2).

### 3.2 Mitochondrial ROS regulation in the peri‐implant microenvironment

**FIGURE 3:**
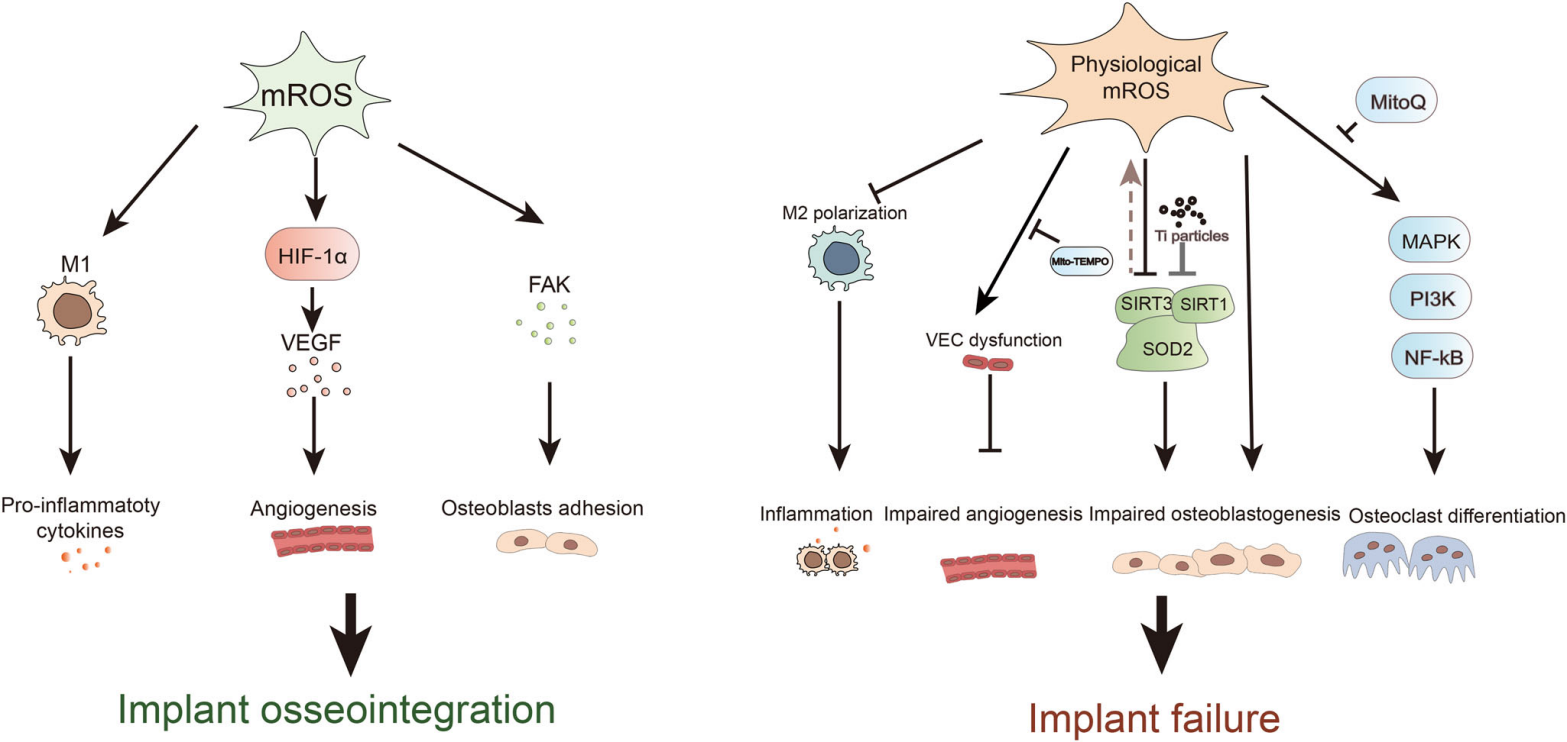
Physiological and pathological regulation in the peri‐implant microenvironment. Mitochondrial reactive oxygen species (mROS) play a crucial physiological role during osseointegration, including M1 macrophage polarization, angiogenesis, osteoblast adhesion and osteoclast differentiation. In pathological situations, excessive mROS have been associated with implant failure. These mROS have been regarded as a master switch for the activation of M2 macrophage polarization and vascular endothelial cell (VEC) dysfunction. Furthermore, the mROS‐related oxidative stress will impair bone homeostasis by impairing mitochondrial antioxidant enzymes. Abbreviations: FAK, focal adhesion kinase; HIF‐1α, hypoxia‐inducible factor‐1α; PKC, protein kinase C; SIRT, sirtuin; SOD, superoxide dismutase; VEGF, vascular endothelial growth factor.

Mitochondrial ROS are tightly regulated signals in the peri‐implant microenvironment with an important role in physiological settings. In the initial phase of implant integration, mROS and the inflammatory phase are interrelated. Generation of mROS has been considered central to determining the inflammatory phenotype of macrophages and stimulating the release of pro‐inflammatory cytokines (Mills et al., 2016a, 2016b; Naik & Dixit, 2011). Moreover, in the hypoxic peri‐implant microenvironment, the released mROS will stabilize hypoxia‐inducible factor‐1α protein, triggering a transcriptional pathway that regulates genes involved in angiogenesis (Reichard & Asosingh, 2019; Terheyden et al., 2012). In the bone remodelling phase, a mild level of ROS has a positive stimulatory effect on bone formation. Mitochondrial ROS might regulate osteoblast adhesion via modulating focal adhesion kinase (FAK) phosphorylation, which is a decisive factor in the success of contact osteogenesis (Rossi et al., 2017; Terheyden et al., 2012; Figure 3).

However, excessive mROS accumulation in the environment has been linked to the development of peri‐implantitis and implant failure in pathophysiological states (Tao, Ge et al., 2020). Recent studies on physiological mROS have focused on diabetic implantitis and aseptic loosening.

As a major risk factor for peri‐implantitis and implant failure, diabetes mellitus creates a hyperglycaemic peri‐implant microenvironment. Studies have identified that hyperglycaemia‐induced excessive mROS generation plays an important role in the pathogenesis of diabetes and its complications. To determine the role of mROS in implant failure in diabetes patients, Wang, Yang et al. (2020) showed that the level of mitochondrial 8‐hydroxy‐2′‐deoxyguanosine, a biomarker of mitochondrial DNA oxidative damage, in the tissue around implants was significantly higher in a diabetic milieu than in a normal milieu. This suggests that mROS might play a crucial role in diabetes‐induced impaired osteointegration. In the detrimental microenvironment, uncontrolled mROS put macrophages into a ‘pseudo‐anoxic’ state, leading to local immunological dysfunction, in which macrophages polarize to M1 (He et al., 2021). The M1 macrophage mitochondria overproduce mROS, causing continuous oxidative stress and a vicious cycle (Mills et al., 2016b). He et al. (2021) reported that a ‘chain armour’ structure (Ce‐TA) implant coating, which mimics the actions of both SOD and catalase, scavenges excess mROS and reshapes the pathological diabetic peri‐implant milieu into a regenerative one, where macrophages are reprogrammed from M1 to M2.

Furthermore, a recent study has shown that mitochondrial dysfunction contributes to VEC dysfunction and inhibition of angiogenesis by increasing ROS production in the peri‐implant microenvironment in diabetes (Hu et al., 2018). In diabetes, VEC mitochondrial fission is triggered by elevated circulating glucose and free fatty acids, leading to oxidative damage to the mitochondrial DNA and mitochondrial membrane hyperpolarization, both of which, in turn, lead to overproduction of ROS (Caja & Enriquez, 2017). In VECs, diabetes‐related mROS overproduction activates subsequent events linked to endothelial dysfunction, such as activation of protein kinase C, impairing angiogenesis, and thus contributes to implant failures (Caja & Enriquez, 2017; Hu et al., 2018). Importantly, VEC dysfunction and impairment of angiogenesis on a titanium surface in the diabetic milieu were both significantly reduced by Mito‐TEMPO (a mitochondria‐targeted ROS antagonist), indicating the importance of mROS in VECs on the implant surface (Hu et al., 2018). Additionally, elevated mROS inhibited osteoblastic differentiation and cell proliferation, enhanced apoptotic damage and compromised osteoblast adherence and morphology, which contributed to the high failure rate of implantation in hyperglycaemia (Takanche et al., 2020). In parallel, Wang, Fu et al. (2021) demonstrated that scavenging mROS of osteoblasts in hyperglycaemic micro‐milieu, via inhibiting the overexpression of Drp1, can rescue osteoblast dysfunction and enhanced in vivo osseointegration was discovered in diabetic rat bone defect models. Overall, excessive mitochondrial oxidative stress in diabetes will cause implant failure in the peri‐implant microenvironment (Figure 3).

The most common long‐term effects of implant surgery are peri‐implant osteolysis and related aseptic loosening, both of which are brought on by metal particles resulting from wear of the implant. The main mechanistic explanations for osteolysis induced by Ti particles are that these particles induce ROS overproduction by impairing mitochondrial respiration (Wang, Fu et al., 2021; Zhu et al., 2021). The mROS‐related oxidative stress will impair bone homeostasis in the peri‐implant microenvironment, leading to implant failure. SIRT family members are NAD^+^‐dependent deacetylases that are essential regulators of mitochondrial oxidative stress by activating mitochondrial SOD in the micro‐milieu around implants. In a concentration‐dependent way, Ti particles reduced the activity of intracellular SOD and other antioxidant enzymes (Zhang, Zhu et al., 2020). Wang, Yang et al. (2020) demonstrated that excessive mROS induced by Ti ions lead to osteoblast autophagy through the mitochondrial sirtuin 3 (SIRT3)–SOD2 pathway. Meanwhile, mROS also decrease osteogenic potential in MSCs by inhibiting the expression of SIRT, and the use of a mitochondrial oxidation scavenger can rescue the Ti‐induced osteolysis via upregulating SIRT1 and increasing SOD2 activity (Zhang, Zhu et al., 2020).

Aseptic loosening is mostly caused by osteoclast‐mediated bone resorption (Zhang, Haddouti et al., 2020). Unfortunately, little research has been published on the direct interaction between osteoclasts and wear particles. Zhang et al. (2021) showed that mROS are involved in osteoclast differentiation via mitogen‐activated protein kinase, phosphoinositide 3‐kinase and nuclear factor‐κB pathways, which can be inhibited by a mitochondria‐targeted antioxidant, MitoQ. However, the involvement of mROS in this process of osseointegration is not clear (Figure 2).

In summary, mROS are central regulators in the peri‐implant microenvironment linked to health and disease processes. However, the mechanisms by which mROS increase implant failure in other pathological conditions, such as hypertension and osteoporosis, have not been well studied. Future research should be directed at exploring the specific mechanism of mROS in the peri‐implant microenvironment in a physiological and pathological state, to discover efficient methods of controlling osseointegration in the peri‐implant microenvironment.

## 4 MITOCHONDRIAL METABOLISM IN THE PERI‐IMPLANT MICROENVIRONMENT

Mitochondrial metabolism is crucial for peri‐implant microenvironmental cell proliferation, differentiation and biological activity. Oxidative phosphorylation and glycolysis are the two main mechanisms for creating ATP in mitochondria. In this section, we discuss the impact that mitochondrial metabolic changes can have in the peri‐implant microenvironment.

Macrophage‐related inflammation is highly relevant for implant integration and long‐term stability based on the polarization of the M1–M2 phenotype, which is associated with a metabolic shift. Macrophages can reprogram their metabolism and function in response to peri‐implant circumstances and stimuli (Curi et al., 2017). In the initial inflammatory phase, M1‐polarized macrophages accelerate pro‐inflammatory responses by increasing glycolysis. The activation of M2 macrophages is accompanied by the induction of mitochondrial biogenesis and OXPHOS by elevating the expression of PGC1β for tissue repair during osseointegration (Mills & O'Neill, 2016; Yao et al., 2021). In line with this, inhibition of mitochondrial oxidative respiration can prevent macrophage polarization to the M2 phenotype (Van den Bossche et al., 2016). Additionally, a recent study in the field of cardiovascular disease indicated that the titanium dioxide nanotubes of Ti implants promote M2 polarization by inhibiting macrophage glycolysis and activating the AMP‐activated protein kinase (AMPK) signalling pathway, and thus producing fewer inflammatory factors and secreting more VEGF for re‐endothelialization (Yu et al., 2021). Therefore, targeting mitochondrial metabolic processes is one way to reprogram macrophages from the M1 to the M2 state to regulate the immune response and promote implant success.

Mesenchymal stem cells adhere to a highly strict metabolic programme in order to meet the high energy demands of differentiation. For example, in an undifferentiated state, MSCs depend mostly on glycolysis. As soon as they begin the osteogenic differentiation process, there is an increase in mitochondrial biogenesis and metabolic flux across the TCA cycle (Simmons & Sikavitsas, 2018). Antimycin A, which inhibits OXPHOS, reduces osteogenic gene expression and decreases extracellular matrix production in MSCs (Brianna et al., 2018), hence mitochondrial oxidative metabolism in MSCs is an important factor affecting osseointegration in the peri‐implant microenvironment. Activation of mitochondrial OXPHOS in osteoprogenitors promotes osteogenesis by acetylating and activating β‐catenin (Brianna et al., 2018). Patients with high MSC oxidative metabolism had more favourable outcomes in osseointegration than those patients with low MSC oxidative metabolism (Shum et al., 2020). Further evidence supporting the role of OXPHOS in osteogenesis is that mice deficient in cyclophilin D (CypD^−/−^) develop more efficient oxidative metabolism in MSCs and show better bone formation than CypD^+/+^ mice (Shum et al., 2020). Moreover, the nicotinamide phosphoribosyltransferase inhibitor FK866 reduces NAD^+^ levels in bone marrow stem cells, which reduces osteogenesis by impairing mitochondrial OXPHOS activity, and thus impairs bone repair (Li et al., 2022). Although, during osteoblast maturation, mitochondrial activity and respiration are diminished, recent studies have shown that mitochondria can interact with the aerobic glycolysis in osteoblasts. Lee et al. (2020) indicated that the mitochondrial malic enzyme is important for sustaining the glycolytic flux in osteoblasts.

In light of these studies, upregulation of mitochondrial OXPHOS activity might contribute to promotion of osseointegration. Hollenberg et al. (2020) showed that upregulating mitochondrial OXPHOS activity by systemic administration of a glycolytic inhibitor can improve bone biomechanical properties in both young and old mice.

However, from the perspective of osteoclasts, increasing mitochondrial OXPHOS in osteoclasts might accelerate osteoclastogenesis and result in bone loss. Osteoclast differentiation is accompanied by increased energy production via enhancing oxygen consumption rate and OXPHOS (Da et al., 2021; Li et al., 2020). Kim et al. (2021) demonstrated that galactic cosmic rays induced increased circulating osteoclast differentiation markers and osteoclast formation by enhancing OXPHOS and thus induced trabecular bone loss in mice. Moreover, Kim et al. (2020) indicated that inhibiting mitochondrial respiration might reduce the number of osteoclasts by inducing Bak and Bax‐dependent mitochondrial apoptosis in early osteoclast progenitors. Consistent with this, the deletion of oxidoreductase iron‐sulfur protein 4 (NDUFS4), which is essential for mitochondrial complex I assembly, provides resistance to inflammation‐induced bone resorption by both osteoclast intrinsic and metabolic/systemic regulation (Jin et al., 2014). Inhibitors of the mitochondrial complex of OXPHOS, such as rotenone, antimycin A and oligomycin, arrest the capacity of RANKL to induce osteoclastogenesis, hence preventing bone loss (Zhang et al., 2018). However, these studies focused mainly on inhibiting osteoclastogenesis, neglecting the osteoclast–osteoblast interaction. Wang, Yu et al. (2021) suggest that enhancing osteoclastogenesis along with robust secretion of transforming growth factor‐β1 significantly promote bone implant integration. Increasing osteoclastogenesis by enhancing OXPHOS in the physiological range might enhance osseointegration, which needs further studies.

**FIGURE 4:**
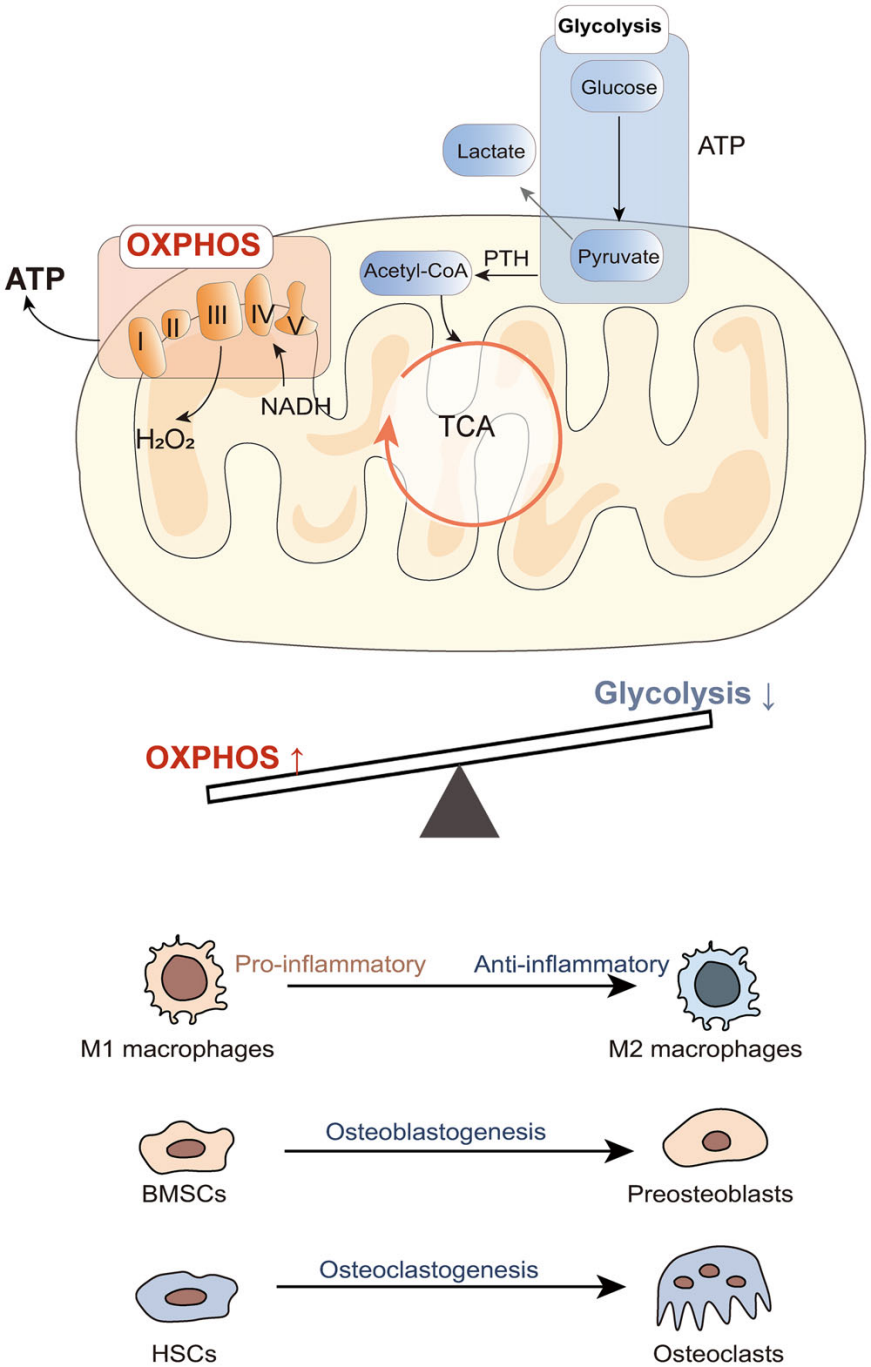
Diagrammatic summary of mitochondrial metabolism during osseointegration in the peri‐implant microenvironment.

In summary, promoting oxidative metabolism in macrophages, osteogenic cells and osteoclasts can increase M2‐polarized macrophages and osteoblastogenesis, all of which are important physiological processes of implant integration. Although enhanced mitochondrial OXPHOS in osteoclasts can induce bone loss in pathological status (Kim et al., 2021; Richardson et al., 2022), osteoclasts can also release growth factors to induce osteoblastogenesis and angiogenesis in the bone microenvironment (Durdan et al., 2022; Han et al., 2018; Wang, Yu et al., 2021). In the resorption phase of bone remodelling, osteoblasts suppress osteoclast production, and M2 macrophages also inhibit osteoclastogenesis. Therefore, we hypothesize that increased mitochondrial OXPHOS might contribute to modification of the peri‐implant microenvironment into a regenerative one for osseointegration in the late bone remodelling phase (Figure 4). However, the peri‐implant microenvironment is a complex structural and biological system that contains different types of cells, and the metabolic regulators are different in peri‐implant cells. How to use mitochondrial metabolism to promote osseointegration in the peri‐implant microenvironment needs further work.

The increased mitochondrial OXPHOS has been observed to be functional in M2‐polarized macrophages, osteoblastogenesis and osteoclastogenesis, which might improve implant osseointegration.

## 5 MITOCHONDRIAL THERAPEUTIC STRATEGIES FOR IMPROVING IMPLANT INTEGRATION

Considering the central role of mitochondrial metabolism and mROS in regulating the behaviours of peri‐implant cells, therapeutic strategies targeting mitochondrial oxidative suppression increasingly show efficacy to improve osseointegration.

Mito‐TEMPO, a mitochondrial antioxidant, has been shown to reverse dysfunction of VECs and impairment of angiogenesis on the Ti surface in hyperglycaemic conditions (Hu et al., 2018). Moreover, it also improves cell activity by reducing mROS in a Ti ion damage osteoblast cell model (Wang, Yang et al., 2020). However, little in vivo study has been performed on Mito‐TEMPO administration to improve bone formation or bone healing. These in vitro findings still suggest mitochondrial oxidative modulation as a promising strategy for improving implant integration.

Given that the surface morphology of implants can modulate the cellular responses, many researchers focus on modification of the implant surface to improve osteointegration. A potent Ce‐TA implant coating has been designed to mimic the actions of both SOD and catalase and scavenge excess mROS, resulting in reshaping the pathological diabetic peri‐implant milieu into a regenerative one. Furthermore, Ce‐TA enhances bone integration of intra‐osseous implants in diabetic rats (He et al., 2021). Consistent with the perspective of immunology, Hang et al. (2022) constructed nanopores on the micro‐pitted Ti surface to promote switching of macrophages from M1 to the M2 form to improve osseointegration by reducing mROS. These findings open a new window for the design of implant surfaces from the perspective of mitochondrial oxidative modulation.

Reagents capable of mitochondrial oxidative suppression, such as adiponectin, ipriflavone and resveratrol (Corrêa et al., 2021; Hua et al., 2020; Tao, Zhou et al., 2020), can improve osseointegration in different animal models. Adiponectin, a fat‐derived adipokine with anti‐diabetic efficacy, can reverse ROS overproduction and alleviate mitochondrial damage by activating AMPK, thus reversing osteoblast impairment and improving the osteointegration (Hu et al., 2017). Chen, Li, Shi et al. (2022) revealed that ipriflavone reduced inflammasome activation in macrophages by decreasing mROS and thus ameliorated the host inflammatory response and promoted early bone healing. Resveratrol can also increase mitochondrial biogenesis and reduce mROS and thus improve mitochondrial function (Ma et al., 2017; Ungvari et al., 2011). In an experimental periodontitis model, resveratrol reduced alveolar bone loss by alleviating mROS levels (Bhattarai et al., 2016). Nevertheless, little is known about the mitochondrial mechanism of resveratrol in the peri‐implant microenvironment.

Although the role of mitochondrial metabolism in the cellular biological events of osteointegration is recognized, there is still a lack of studies on therapeutic strategies targeting mitochondrial metabolism to improve osseointegration. Notably, in mitochondria‐targeted therapeutic studies, the regulation of mROS and mitochondrial metabolism are closely related. Inhibition of mROS can also increase OXPHOS by enhancing mitochondrial function (Hu et al., 2017). Therefore, endogenous molecular targets that optimize mitochondrial function are potential targets for improving osseointegration.

Sirtuin 3, which is mostly found in mitochondria, has a significant impact on mitochondrial homeostasis and mitochondrial oxidative stress (Huang et al., 2022). Honokiol, a SIRT3 agonist, has the potential to improve implant osseointegration in patients with diabetes, because it might help to reverse the negative effects of diabetes on bone repair (Huang et al., 2022). Wang, Yang et al. (2020) showed that SOD2 acetylation generated by Ti ions might be repaired by overexpression of SIRT3, which decreases the acetylation of SOD2 and increases SOD2 activity, therefore preserving the stability of mitochondrial oxygen free radicals and reversing autophagy induced by Ti ions. Therefore, osteoblastic cell damage induced by Ti ions might be caused, in part, by SIRT3/SOD2 autophagy. Paradoxically, osteoclast activation and inflammatory cytokine production are both suppressed when SIRT3 is inhibited, which, in turn, reduces Ti particle‐induced bone loss. Osseointegration is a complex biological process that requires interactions among various cell types, including osteoclasts and osteoblasts. Future study of the involvement of SIRT3 in the pri‐implant microenvironment is therefore necessary.

Cyclophilin D is a crucial regulator of the mitochondrial permeability transition pore (Murphy, 2022). It is possible to investigate the cellular dependence on mitochondria‐derived ATP and metabolites by manipulating CypD. Smith et al. (2020) found that CypD knockout mice had considerably more osteoblast activity, bone formation and improved biomechanical qualities in comparison to control animals during fracture healing. Therefore, inhibition of CypD by NIM811, Debio025 or JW47 might enhance bone growth and implant integration.

Drp1 is the main pro‐fission protein that regulates mitochondrial shape, which is also closely related to mROS production and mitochondrial metabolism (Fan et al., 2022; Liu, Zhao et al., 2020). Recent studies have provided new insights into the role of the Drp1–ROS‐dependent mitochondrial pathway in osseointegration, especially in diabetes mellitus. Excessive ROS‐induced mitochondrial fragmentation mediated by diabetes compromises implant osteointegration. Shi et al. (2021) showed that pharmacological inhibition of Drp1 reduced periodontal cell death, inflammatory responses and alveolar bone loss. Wang, Fu et al. (2021) showed that Zn^2+^ and Sr^2^^+^ released from ZnO and Sr(OH)_2_ coated implants can restore mitochondrial dysfunction in hyperglycaemia by downregulation of *Drp1* gene expression, ultimately enhancing osseointegration. Drp1 has been shown to offer significant therapeutic promise for the treatment of peri‐implantitis based on these findings.

Targeting mitochondrial endogenous molecular pathways and enhancing mitochondrial function could be a new approach for implant treatment in the future. However, the underlying molecular mechanisms of the drugs need further elucidation to identify the mitochondrial regulation in the peri‐implant microenvironment.

## 6 CONCLUSION

**FIGURE 5:**
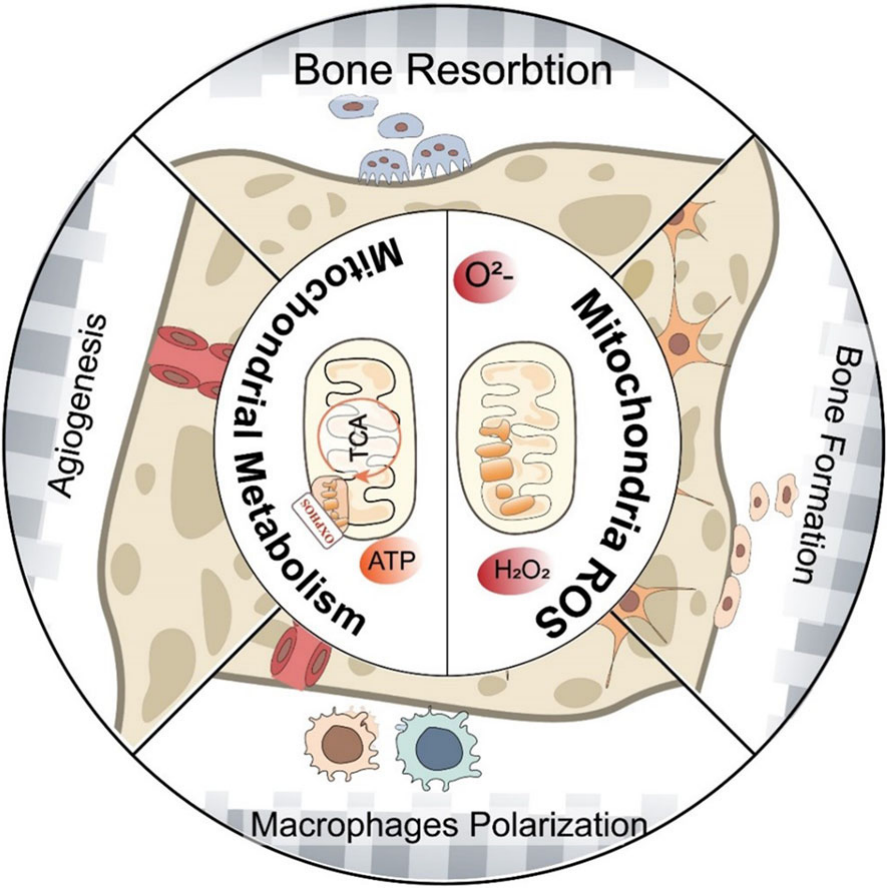
Mitochondrial regulation in the peri‐implant microenvironment. Mitochondrial regulation in the peri‐implant milieu, includes the regulation of mROS and mitochondrial metabolism in angiogenesis, the polarization of macrophage immune responses, bone formation and bone resorption during osseointegration.

In recent years, it has been discovered that mitochondria have a significant impact on physiological processes in peri‐implant cells, pathological alterations and therapeutic outcomes. We have summarized the most important results establishing the role and molecular control of mitochondria in the peri‐implant milieu (Figure 5). Although we focused on the control of mitochondrial metabolism and mROS in the peri‐implant milieu, mitochondrial regulation is not restricted to one component; mitochondrial dynamics and mitochondrial biogenesis are also involved and interact (Sokolova, 2018). Although much progress has been achieved, many questions remain.

First, research on the mechanism of osseointegration based on the mitochondrial regulation of osteoclasts and VECs is still seriously insufficient. A new approach is to manipulate mitochondria in regulating the coupling effect of osteoclasts, VECs, osteoblasts and macrophages.

Second, more research is needed to explore the mitochondrial metabolism‐targeted therapeutic strategies for improving osseointegration. Enhancing OXPHOS might be a promising target in implant therapy. More work is needed on how to increase mitochondria by reagents or implant surface modification.

Third, current treatments are limited to precise manipulation of mitochondria in a cell type‐ and space–time‐specific manner, because implant osseointegration is a biological process involving various cell types.

Finally, most of the studies that focus on mitochondrial regulation in osseointegration are in vitro studies. However, Ti implants are placed clinically in the complex peri‐implant microenvironment; hence, further research into the intricate relationship between mitochondria and implant osseointegration in vivo is urgently required. A comprehensive understanding is required to combine the mitochondrial regulation of individual cells.

## AUTHOR CONTRIBUTIONS

All authors conceived and designed the manuscript, prepared the figures, drafted the manuscript, and edited and revised the manuscript. All authors have read and approved the final version of this manuscript and agree to be accountable for all aspects of the work in ensuring that questions related to the accuracy or integrity of any part of the work are appropriately investigated and resolved. All persons designated as authors qualify for authorship, and all those who qualify for authorship are listed.

## CONFLICT OF INTEREST

None declared.

## References

[eph13297-bib-0001] Agidigbi, T. S. , & Kim, C. (2019). Reactive oxygen species in osteoclast differentiation and possible pharmaceutical targets of ROS‐mediated osteoclast diseases. International Journal of Molecular Sciences, 20(14), 3576. 31336616 10.3390/ijms20143576 PMC6678498

[eph13297-bib-0002] Amengual‐Peñafiel, L. , Córdova, L. A. , Constanza Jara‐Sepúlveda, M. , Brañes‐Aroca, M. , Marchesani‐Carrasco, F. , & Cartes‐Velásquez, R. (2021). Osteoimmunology drives dental implant osseointegration: A new paradigm for implant dentistry. Japanese Dental Science Review, 57, 12–19. 33737990 10.1016/j.jdsr.2021.01.001 PMC7946347

[eph13297-bib-0003] Bahney, C. S. , Zondervan, R. L. , Allison, P. , Theologis, A. , Ashley, J. W. , Ahn, J. , Miclau, T. , Marcucio, R. S. , & Hankenson, K. D. (2019). Cellular biology of fracture healing. Journal of Orthopaedic Research, 37(1), 35–50. 30370699 10.1002/jor.24170 PMC6542569

[eph13297-bib-0004] Bennett, C. F. , Latorre‐Muro, P. , & Puigserver, P. (2022). Mechanisms of mitochondrial respiratory adaptation. Nature Reviews Molecular Cell Biology, 23(12), 817–835. 35804199 10.1038/s41580-022-00506-6 PMC9926497

[eph13297-bib-0005] Bhattarai, G. , Poudel, S. B. , Kook, S.‐H. , & Lee, J.‐C. (2016). Resveratrol prevents alveolar bone loss in an experimental rat model of periodontitis. Acta Biomaterialia, 29, 398–408. 26497626 10.1016/j.actbio.2015.10.031

[eph13297-bib-0006] Blajszczak, C. , & Bonini, M. G. (2017). Mitochondria targeting by environmental stressors: Implications for redox cellular signaling. Toxicology, 391, 84–89. 28750850 10.1016/j.tox.2017.07.013 PMC5939563

[eph13297-bib-0007] Borys, J. , Maciejczyk, M. , Antonowicz, B. , Sidun, J. , Świderska, M. , & Zalewska, A. (2019). Free radical production, inflammation and apoptosis in patients treated with titanium mandibular fixations—An observational study [Original research]. Frontiers in Immunology, 10, 2662. 31781128 10.3389/fimmu.2019.02662 PMC6857478

[eph13297-bib-0008] Brand, M. D. (2016). Mitochondrial generation of superoxide and hydrogen peroxide as the source of mitochondrial redox signaling. Free Radical Biology and Medicine, 100, 14–31. 27085844 10.1016/j.freeradbiomed.2016.04.001

[eph13297-bib-0009] Brand, M. D. (2020). Riding the tiger ‐ physiological and pathological effects of superoxide and hydrogen peroxide generated in the mitochondrial matrix. Critical Reviews in Biochemistry and Molecular Biology, 55(6), 592–661. 33148057 10.1080/10409238.2020.1828258

[eph13297-bib-0010] Bressan, E. , Ferroni, L. , Gardin, C. , Bellin, G. , Sbricoli, L. , Sivolella, S. , Brunello, G. , Schwartz‐Arad, D. , Mijiritsky, E. , Penarrocha, M. , Penarrocha, D. , Taccioli, C. , Tatullo, M. , Piattelli, A. , & Zavan, B. (2019). Metal nanoparticles released from dental implant surfaces: Potential contribution to chronic inflammation and peri‐implant bone loss. Materials, 12(12), 2036. 31242601 10.3390/ma12122036 PMC6630980

[eph13297-bib-0011] Brianna, H. , Shares, M. , Busch, M. , White, N. , Shum, L. , & Eliseev, R. A. (2018). Active mitochondria support osteogenic differentiation by stimulating β‐catenin acetylation. Journal of Biological Chemistry, 293(41), 16019–16027. 30150300 10.1074/jbc.RA118.004102 PMC6187642

[eph13297-bib-0012] Caja, S. , & Enriquez, J. (2017). Mitochondria in endothelial cells: Sensors and integrators of environmental cues. Redox Biology, 12, 821–827. 28448943 10.1016/j.redox.2017.04.021 PMC5406579

[eph13297-bib-0013] Chakrabarty, R. P. , & Chandel, N. S. (2022). Beyond ATP, new roles of mitochondria. The Biochemist, 44(4), 2–8. 36248614 10.1042/bio_2022_119 PMC9558425

[eph13297-bib-0014] Chen, M. , Li, M. , Wei, Y. , Xue, C. , Chen, M. , Fei, Y. , Tan, L. , Luo, Z. , Cai, K. , & Hu, Y. (2022). ROS‐activatable biomimetic interface mediates in‐situ bioenergetic remodeling of osteogenic cells for osteoporotic bone repair. Biomaterials, 291, 121878. 36335716 10.1016/j.biomaterials.2022.121878

[eph13297-bib-0015] Chen, M. , Wang, D. , Li, M. , He, Y. , He, T. , Chen, M. , Hu, Y. , Luo, Z. , & Cai, K. (2022). Nanocatalytic biofunctional MOF coating on titanium implants promotes osteoporotic bone regeneration through cooperative Pro‐osteoblastogenesis MSC reprogramming. ACS Nano, 16(9), 15397–15412. 36106984 10.1021/acsnano.2c07200

[eph13297-bib-0016] Chen, W. , Zhu, W.‐Q. , & Qiu, J. (2021). Impact of exogenous metal ions on peri‐implant bone metabolism: A review. RSC Advances, 11(22), 13152–13163. 35423842 10.1039/d0ra09395e PMC8697588

[eph13297-bib-0017] Chen, Y. , Li, J. , Shi, J. , Ning, D. , Feng, J. , Lin, W. , He, F. , & Xie, Z. (2022). Ipriflavone suppresses NLRP3 inflammasome activation in host response to biomaterials and promotes early bone healing. Journal of Clinical Periodontology, 49(8), 814–827. 35569032 10.1111/jcpe.13647

[eph13297-bib-0018] Corrêa, M. G. , Ribeiro, F. V. , Pimentel, S. P. , Benatti, B. B. , Felix Silva, P. H. , Casati, M. Z. , & Cirano, F. R. (2021). Impact of resveratrol in the reduction of the harmful effect of diabetes on peri‐implant bone repair: Bone‐related gene expression, counter‐torque and micro‐CT analysis in rats. Acta Odontologica Scandinavica, 79(3), 174–181. 32697922 10.1080/00016357.2020.1797159

[eph13297-bib-0019] Curi, R. , de Siqueira Mendes, R. , de Campos Crispin, L. A. , Norata, G. D. , Sampaio, S. C. , & Newsholme, P. (2017). A past and present overview of macrophage metabolism and functional outcomes. Clinical Science, 131(12), 1329–1342. 28592702 10.1042/CS20170220

[eph13297-bib-0020] Da, W. , Tao, L. , & Zhu, Y. (2021). The role of osteoclast energy metabolism in the occurrence and development of osteoporosis. Frontiers in Endocrinology, 12, 556. 10.3389/fendo.2021.675385 PMC8150001 34054735

[eph13297-bib-0021] Davidson, S. M. , & Duchen, M. R. (2007). Endothelial mitochondria. Circulation Research, 100(8), 1128–1141. 17463328 10.1161/01.RES.0000261970.18328.1d

[eph13297-bib-0022] Diomede, F. , Marconi, G. D. , Fonticoli, L. , Pizzicanella, J. , Merciaro, I. , Bramanti, P. , Mazzon, E. , & Trubiani, O. (2020). Functional relationship between osteogenesis and angiogenesis in tissue regeneration. International Journal of Molecular Sciences, 21(9), 3242. 32375269 10.3390/ijms21093242 PMC7247346

[eph13297-bib-0023] Durdan, M. M. , Azaria, R. D. , & Weivoda, M. M. (2022). Novel insights into the coupling of osteoclasts and resorption to bone formation. Seminars in Cell & Developmental Biology, 123, 4–13. 34756783 10.1016/j.semcdb.2021.10.008 PMC8840962

[eph13297-bib-0024] Fan, K. , Ding, X. , Zang, Z. , Zhang, Y. , Tang, X. , Pei, X. , Chen, Q. , Yin, H. , Zheng, X. , Chen, Y. , Li, S. , & Yang, H. (2022). Drp1‐Mediated mitochondrial metabolic dysfunction inhibits the tumor growth of pituitary adenomas. Oxidative Medicine and Cellular Longevity, 2022, 1. 10.1155/2022/5652586 PMC8967574 35368865

[eph13297-bib-0025] Fuhrmann, D. C. , & Brüne, B. (2017). Mitochondrial composition and function under the control of hypoxia. Redox Biology, 12, 208–215. 28259101 10.1016/j.redox.2017.02.012 PMC5333533

[eph13297-bib-0026] Fukai, T. , & Ushio‐Fukai, M. J. C. (2020). Cross‐talk between NADPH oxidase and mitochondria: Role in ROS signaling and angiogenesis. Cells, 9(8), 1849. 32781794 10.3390/cells9081849 PMC7466096

[eph13297-bib-0027] Ghensi, P. , Bressan, E. , Gardin, C. , Ferroni, L. , Ruffato, L. , Caberlotto, M. , Soldini, C. , & Zavan, B. (2017). Osteo growth induction titanium surface treatment reduces ROS production of mesenchymal stem cells increasing their osteogenic commitment. Materials Science and Engineering: C, 74, 389–398. 28254309 10.1016/j.msec.2016.12.032

[eph13297-bib-0028] Gu, D. R. , Lee, J. N. , Oh, G.‐S. , Kim, H. J. , Kim, M. S. , & Lee, S. H. (2017). The inhibitory effect of beta‐lapachone on RANKL‐induced osteoclastogenesis. Biochemical and Biophysical Research Communications, 482(4), 1073–1079. 27913299 10.1016/j.bbrc.2016.11.160

[eph13297-bib-0029] Guo, D. , Wang, Q. , Li, C. , Wang, Y. , & Chen, X. (2017). VEGF stimulated the angiogenesis by promoting the mitochondrial functions. Oncotarget, 8(44), 77020–77027. 29100366 10.18632/oncotarget.20331 PMC5652760

[eph13297-bib-0030] Hadjidakis, D. J. , & Androulakis, I. I. (2006). Bone remodeling. Annals of the New York Academy of Sciences, 1092(1), 385–396. 17308163 10.1196/annals.1365.035

[eph13297-bib-0031] Han, Y. , You, X. , Xing, W. , Zhang, Z. , & Zou, W. (2018). Paracrine and endocrine actions of bone—The functions of secretory proteins from osteoblasts, osteocytes, and osteoclasts. Bone Research, 6(1), 16. 29844945 10.1038/s41413-018-0019-6 PMC5967329

[eph13297-bib-0032] Hang, R. , Zhao, Y. , Zhang, Y. , Yao, R. , Yao, X. , Sun, Y. , Huang, D. , & Hang, R. (2022). The role of nanopores constructed on the micropitted titanium surface in the immune responses of macrophages and the potential mechanisms. Journal of Materials Chemistry B, 10(38), 7732–7743. 36069532 10.1039/d2tb01263d

[eph13297-bib-0033] He, Q. , Yuan, S. , Tang, H. , Wang, S. , Mu, Z. , Li, D. , Wang, S. , Jing, X. , Hu, S. , Ji, P. , & Chen, T. (2021). Safeguarding osteointegration in diabetic patients: A potent “Chain armor” coating for scavenging ROS and macrophage reprogramming in a microenvironment‐responsive manner. Advanced Functional Materials, 31(31), 2101611.

[eph13297-bib-0034] He, Y. , Gao, Y. , Ma, Q. , Zhang, X. , Zhang, Y. , & Song, W. (2022). Nanotopographical cues for regulation of macrophages and osteoclasts: Emerging opportunities for osseointegration. Journal of Nanobiotechnology, 20(1), 510. 36463225 10.1186/s12951-022-01721-1 PMC9719660

[eph13297-bib-0035] Hollenberg, A. M. , Smith, C. O. , Shum, L. C. , Awad, H. , & Eliseev, R. A. (2020). Lactate dehydrogenase inhibition with oxamate exerts bone anabolic effect [Dec]. Journal of Bone and Mineral Research, 35(12), 2432–2443. 32729639 10.1002/jbmr.4142 PMC7736558

[eph13297-bib-0036] Hu, K. , & Olsen, B. R. (2016). The roles of vascular endothelial growth factor in bone repair and regeneration. Bone, 91, 30–38. 27353702 10.1016/j.bone.2016.06.013 PMC4996701

[eph13297-bib-0037] Hu, X.‐F. , Wang, L. , Lu, Y.‐Z. , Xiang, G. , Wu, Z.‐X. , Yan, Y.‐B. , Zhang, Y. , Zhao, X. , Zang, Y. , Shi, L. , Lei, W. , & Feng, Y.‐F. (2017). Adiponectin improves the osteointegration of titanium implant under diabetic conditions by reversing mitochondrial dysfunction via the AMPK pathway in vivo and in vitro. Acta Biomaterialia, 61, 233–248. 28624657 10.1016/j.actbio.2017.06.020

[eph13297-bib-0038] Hu, X.‐F. , Wang, L. , Xiang, G. , Lei, W. , & Feng, Y.‐F. (2018). Angiogenesis impairment by the NADPH oxidase‐triggered oxidative stress at the bone‐implant interface: Critical mechanisms and therapeutic targets for implant failure under hyperglycemic conditions in diabetes. Acta Biomaterialia, 73, 470–487. 29649637 10.1016/j.actbio.2018.04.008

[eph13297-bib-0039] Hua, Y. , Bi, R. , Li, Z. , & Li, Y. (2020). Resveratrol treatment promotes titanium implant osseointegration in diabetes mellitus rats. Journal of Orthopaedic Research, 38(10), 2113–2119. 32141632 10.1002/jor.24651

[eph13297-bib-0040] Huang, X. , Shu, H. , Ren, C. , & Zhu, J. (2022). SIRT3 improves bone regeneration and rescues diabetic fracture healing by regulating oxidative stress. Biochemical and Biophysical Research Communications, 604, 109–115. 35303676 10.1016/j.bbrc.2022.03.001

[eph13297-bib-0041] Jetten, N. , Verbruggen, S. , Gijbels, M. J. , Post, M. J. , De Winther, M. P. J. , & Donners, M. M. P. C. (2014). Anti‐inflammatory M2, but not pro‐inflammatory M1 macrophages promote angiogenesis in vivo. Angiogenesis, 17(1), 109–118. 24013945 10.1007/s10456-013-9381-6

[eph13297-bib-0042] Jin, Z. , Wei, W. , Yang, M. , Du, Y. , & Wan, Y. (2014). Mitochondrial complex i activity suppresses inflammation and enhances bone resorption by shifting macrophage‐osteoclast polarization. Cell Metabolism, 20(3), 483–498. 25130399 10.1016/j.cmet.2014.07.011 PMC4156549

[eph13297-bib-0043] Kim, H. N. , Ponte, F. , Nookaew, I. , Ucer Ozgurel, S. , Marques‐Carvalho, A. , Iyer, S. , Warren, A. , Aykin‐Burns, N. , Krager, K. , Sardao, V. A. , Han, L. , de Cabo, R. , Zhao, H. , Jilka, R. L. , Manolagas, S. C. , & Almeida, M. (2020). Estrogens decrease osteoclast number by attenuating mitochondria oxidative phosphorylation and ATP production in early osteoclast precursors. Scientific Reports, 10(1), 11933. 32686739 10.1038/s41598-020-68890-7 PMC7371870

[eph13297-bib-0044] Kim, H. N. , Richardson, K. K. , Krager, K. J. , Ling, W. , Simmons, P. , Allen, A. R. , & Aykin‐Burns, N. (2021). Simulated galactic cosmic rays modify mitochondrial metabolism in osteoclasts, increase osteoclastogenesis and cause trabecular bone loss in mice. International Journal of Molecular Sciences, 22(21), 11711. 34769141 10.3390/ijms222111711 PMC8583929

[eph13297-bib-0045] Kubatzky, K. F. , Uhle, F. , & Eigenbrod, T. (2018). From macrophage to osteoclast – How metabolism determines function and activity. Cytokine, 112, 102–115. 29914791 10.1016/j.cyto.2018.06.013

[eph13297-bib-0046] Kumar, S. , Prithviraj, D. , Musaib Syed, D. , Varne, S. S. , Khan, F. A. , & Aliya, S. (2021). Healing of wound after implant placement – A review. Annals of the Romanian Society for Cell Biology, 25(5), 3133–3140.

[eph13297-bib-0047] Lampropoulou, V. , Sergushichev, A. , Bambouskova, M. , Nair, S. , Vincent, E. E. , Loginicheva, E. , Cervantes‐Barragan, L. , Ma, X. , Ching‐Cheng Huang, S. , Griss, T. , Weinheimer, C. J. , Khader, S. , Randolph, G. J. , Pearce, E. J. , Jones, R. G. , Diwan, A. , Diamond, M. S. , & Artyomov, M. N. (2016). Itaconate links inhibition of succinate dehydrogenase with macrophage metabolic remodeling and regulation of inflammation. Cell Metabolism, 24(1), 158–166. 27374498 10.1016/j.cmet.2016.06.004 PMC5108454

[eph13297-bib-0048] Lee, W.‐C. , Ji, X. , Nissim, I. , & Long, F. (2020). Malic enzyme couples mitochondria with aerobic glycolysis in osteoblasts. Cell Reports, 32(10), 108108. 32905773 10.1016/j.celrep.2020.108108 PMC8183612

[eph13297-bib-0049] Lemma, S. , Sboarina, M. , Porporato, P. E. , Zini, N. , Sonveaux, P. , Di Pompo, G. , Baldini, N. , & Avnet, S. (2016). Energy metabolism in osteoclast formation and activity. The International Journal of Biochemistry & Cell Biology, 79, 168–180. 27590854 10.1016/j.biocel.2016.08.034

[eph13297-bib-0050] Li, B. , Lee, W.‐C. , Song, C. , Ye, L. , Abel, E. D. , & Long, F. (2020). Both aerobic glycolysis and mitochondrial respiration are required for osteoclast differentiation. The FASEB Journal, 34(8), 11058–11067. 32627870 10.1096/fj.202000771R

[eph13297-bib-0051] Li, B. , Shi, Y. , Liu, M. , Wu, F. , Hu, X. , Yu, F. , Wang, C. , & Ye, L. (2022). Attenuates of NAD+ impair BMSC osteogenesis and fracture repair through OXPHOS. Stem Cell Research & Therapy, 13(1), 77. 35193674 10.1186/s13287-022-02748-9 PMC8864833

[eph13297-bib-0052] Liu, P.‐S. , & Ho, P.‐C. (2018). Mitochondria: A master regulator in macrophage and t cell immunity. Mitochondrion, 41, 45–50. 29146487 10.1016/j.mito.2017.11.002

[eph13297-bib-0053] Liu, X. , Zhao, X. , Li, X. , Lv, S. , Ma, R. , Qi, Y. , Abulikemu, A. , Duan, H. , Guo, C. , Li, Y. , & Sun, Z. (2020). PM2. 5 triggered apoptosis in lung epithelial cells through the mitochondrial apoptotic way mediated by a ROS‐DRP1‐mitochondrial fission axis. Journal of Hazardous Materials, 397, 122608. 32387827 10.1016/j.jhazmat.2020.122608

[eph13297-bib-0054] Liu, Y. , Rath, B. , Tingart, M. , & Eschweiler, J. (2020). Role of implants surface modification in osseointegration: A systematic review. Journal of Biomedical Materials Research. Part A, 108(3), 470–484. 31664764 10.1002/jbm.a.36829

[eph13297-bib-0055] Ma, Z. , Zhang, Y. , Li, Q. , Xu, M. , Bai, J. , & Wu, S. (2017). Resveratrol improves alcoholic fatty liver disease by downregulating HIF‐1α expression and mitochondrial ROS production. PLoS One, 12(8), e0183426. 28817659 10.1371/journal.pone.0183426 PMC5560649

[eph13297-bib-0056] Mijiritsky, E. , Ferroni, L. , Gardin, C. , Peleg, O. , Gultekin, A. , Saglanmak, A. , Delogu, L. G. , Mitrecic, D. , Piattelli, A. , Tatullo, M. , & Zavan, B. (2020). Presence of ROS in inflammatory environment of peri‐implantitis tissue: In vitro and in vivo human evidence. Journal of Clinical Medicine, 9(1), 38. 10.3390/jcm9010038 PMC7019824 31878038

[eph13297-bib-0057] Mills, E. , Kelly, B. , Logan, A. , Costa, A. , Varma, M. , Bryant, C. , Tourlomousis, P. , Däbritz, J. H. M. , Gottlieb, E. , Latorre, I. , Corr, S. C. , McManus, G. , Ryan, D. , Jacobs, H. T. , Szibor, M. , Xavier, R. J. , Braun, T. , Frezza, C. , Murphy, M. P. , & O'Neill, L. A. (2016a). Repurposing mitochondria from ATP production to ROS generation drives a pro‐inflammatory phenotype in macrophages that depends on succinate oxidation by complex iI. Cell, 167(2), 457. 27667687 10.1016/j.cell.2016.08.064 PMC5863951

[eph13297-bib-0058] Mills, E. L. , Kelly, B. , Logan, A. , Costa, A. S. , Varma, M. , Bryant, C. E. , Tourlomousis, P. , Däbritz, J. H. M. , Gottlieb, E. , Latorre, I. , Corr, S. C. , McManus, G. , Ryan, D. , Jacobs, H. T. , Szibor, M. , Xavier, R. J. , Braun, T. , Frezza, C. , Murphy, M. P. , & O'Neill, L. A. (2016b). Succinate dehydrogenase supports metabolic repurposing of mitochondria to drive inflammatory macrophages. Cell, 167(2), 457–470.e13. e413. 27667687 10.1016/j.cell.2016.08.064 PMC5863951

[eph13297-bib-0059] Mills, E. L. , & O'Neill, L. A. (2016). Reprogramming mitochondrial metabolism in macrophages as an anti‐inflammatory signal. European Journal of Immunology, 46(1), 13–21. 26643360 10.1002/eji.201445427

[eph13297-bib-0060] Mouthuy, P. A. , Snelling, S. J. B. , Dakin, S. G. , Milković, L. , Gašparović, A. , Carr, A. J. , & Žarković, N. (2016). Biocompatibility of implantable materials: An oxidative stress viewpoint. Biomaterials, 109, 55–68. 27669498 10.1016/j.biomaterials.2016.09.010

[eph13297-bib-0061] Murphy, E. (2022). Cyclophilin d regulation of the mitochondrial permeability transition pore. Current Opinion in Physiology, 25, 100486. 35296110 10.1016/j.cophys.2022.100486 PMC8920311

[eph13297-bib-0062] Naik, E. , & Dixit, V. M. (2011). Mitochondrial reactive oxygen species drive proinflammatory cytokine production. Journal of Experimental Medicine, 208(3), 417–420. 21357740 10.1084/jem.20110367 PMC3058577

[eph13297-bib-0063] Oishi, Y. , & Manabe, I. (2018). Macrophages in inflammation, repair and regeneration. International Immunology, 30(11), 511–528. 30165385 10.1093/intimm/dxy054

[eph13297-bib-0064] Palmquist, A. , Omar, O. M. , Esposito, M. , Lausmaa, J. , & Thomsen, P. (2010). Titanium oral implants: Surface characteristics, interface biology and clinical outcome. Journal of the Royal Society, Interface, 7(suppl_5), S515–S527. 20591849 10.1098/rsif.2010.0118.focus PMC2952179

[eph13297-bib-0065] Park‐Min, K.‐H. (2019). Metabolic reprogramming in osteoclasts. Seminars in Immunopathology, 41(5), 565–572. 31552471 10.1007/s00281-019-00757-0 PMC7671717

[eph13297-bib-0066] Pfeiffenberger, M. , Damerau, A. , Lang, A. , Buttgereit, F. , Hoff, P. , & Gaber, T. (2021). Fracture healing research – Shift towards in vitro modeling? Biomedicines, 9(7), 748. 34203470 10.3390/biomedicines9070748 PMC8301383

[eph13297-bib-0067] Qing, J. , Zhang, Z. , Novák, P. , Zhao, G. , & Yin, K. (2020). Mitochondrial metabolism in regulating macrophage polarization: An emerging regulator of metabolic inflammatory diseases. Acta Biochim Biophys Sin (Shanghai), 52(9), 917–926. 32785581 10.1093/abbs/gmaa081

[eph13297-bib-0068] Reichard, A. , & Asosingh, K. (2019). The role of mitochondria in angiogenesis. Molecular Biology Reports, 46(1), 1393–1400. 30460535 10.1007/s11033-018-4488-x PMC6426673

[eph13297-bib-0069] Richardson, K. K. , Ling, W. , Krager, K. , Fu, Q. , Byrum, S. D. , Pathak, R. , Aykin‐Burns, N. , & Kim, H. N. (2022). Ionizing radiation activates mitochondrial function in osteoclasts and causes bone loss in young adult male mice. International Journal of Molecular Sciences, 23(2), 675. 35054859 10.3390/ijms23020675 PMC8775597

[eph13297-bib-0070] Ripszky Totan, A. , Imre, M. M. , Parvu, S. , Meghea, D. , Radulescu, R. , Enasescu, D. S. A. , Moisa, M. R. , & Pituru, S. M. (2022). Autophagy plays multiple roles in the soft‐tissue healing and osseointegration in dental implant surgery – A narrative review. Materials (Basel), 15(17), 6041. 36079421 10.3390/ma15176041 PMC9457242

[eph13297-bib-0071] Rohlenova, K. , Veys, K. , Miranda‐Santos, I. , De Bock, K. , & Carmeliet, P. (2018). Endothelial cell metabolism in health and disease. Trends in Cell Biology, 28(3), 224–236. 29153487 10.1016/j.tcb.2017.10.010

[eph13297-bib-0115] Rossi, M. C. , Bezerra, F. J. B. , Silva, R. A. , Crulhas, B. P. , Fernandes, C. J. C. , Nascimento, A. S. , Pedrosa, V. A. , Padilha, P. , & Zambuzzi, W. F. (2017). Titanium‐released from dental implant enhances pre‐osteoblast adhesion by ROS modulating crucial intracellular pathways. Journal of Biomedical Materials Research. Part A, 105(11), 2968–2976. 28639351 10.1002/jbm.a.36150

[eph13297-bib-0072] Shen, K. , Pender, C. L. , Bar‐Ziv, R. , Zhang, H. , Wickham, K. , Willey, E. , Durieux, J. , Ahmad, Q. , & Dillin, A. (2022). Mitochondria as cellular and organismal signaling hubs. Annual Review of Cell and Developmental Biology, 38(1), 179–218. 10.1146/annurev-cellbio-120420-015303 35804477

[eph13297-bib-0073] Shen, X. , Fang, K. , Ru Yie, K. H. , Zhou, Z. , Shen, Y. , Wu, S. , Zhu, Y. , Deng, Z. , Ma, P. , Ma, J. , & Liu, J. (2022). High proportion strontium‐doped micro‐arc oxidation coatings enhance early osseointegration of titanium in osteoporosis by anti‐oxidative stress pathway. Bioactive Materials, 10, 405–419. 34901556 10.1016/j.bioactmat.2021.08.031 PMC8636681

[eph13297-bib-0074] Shi, L. , Ji, Y. , Zhao, S. , Li, H. , Jiang, Y. , Mao, J. , Chen, Y. , Zhang, X. , Mao, Y. , Sun, X. , Wang, P. , Ma, J. , & Huang, S. (2021). Crosstalk between reactive oxygen species and Dynamin‐related protein 1 in periodontitis. Free Radical Biology and Medicine, 172, 19–32. 34052344 10.1016/j.freeradbiomed.2021.05.031

[eph13297-bib-0075] Shum, L. C. , Hollenberg, A. M. , Baldwin, A. L. , Kalicharan, B. H. , Maqsoodi, N. , Rubery, P. T. , Mesfin, A. , & Eliseev, R. A. (2020). Role of oxidative metabolism in osseointegration during spinal fusion. PLoS ONE, 15(11), e0241998. 33166330 10.1371/journal.pone.0241998 PMC7652281

[eph13297-bib-0076] Shum, L. C. , White, N. s. , Mills, B. N. , Bentley, K. L. , & Eliseev, R. A. (2016). Energy metabolism in mesenchymal stem cells during osteogenic differentiation. Stem Cells and Development, 25(2), 114–122. 26487485 10.1089/scd.2015.0193 PMC4733323

[eph13297-bib-0077] Simmons, A. D. , & Sikavitsas, V. I. (2018). Monitoring bone tissue engineered (BTE) constructs based on the shifting metabolism of differentiating stem cells. Annals of Biomedical Engineering, 46(1), 37–47. 29022110 10.1007/s10439-017-1937-y

[eph13297-bib-0078] Smith, C. O. , Sheu, T.‐J. , Sautchuk Jr., R. , Schilling, K. , Shum, L. C. , Paine, A. , Huber, A. , Gira, E. , Brown, E. , Awad, H. , & Awad, H. J. B. (2020). Inhibition of the mitochondrial permeability transition improves bone fracture repair. Bone, 11(22), 13152. 10.1016/j.bone.2020.115391 PMC7354230 32360587

[eph13297-bib-0079] Sokolova, I. (2018). Mitochondrial adaptations to variable environments and their role in animals’ stress tolerance. Integrative and Comparative Biology, 58(3), 519–531. 29701785 10.1093/icb/icy017

[eph13297-bib-0080] Sun, R. , Bai, L. , Yang, Y. , Ding, Y. , Zhuang, J. , & Cui, J. (2022). Nervous system‐driven osseointegration. International Journal of Molecular Sciences, 23(16), 8893. 36012155 10.3390/ijms23168893 PMC9408825

[eph13297-bib-0081] Takanche, J. S. , Kim, J.‐E. , Han, S.‐H. , & Yi, H.‐K. (2020). Effect of gomisin a on osteoblast differentiation in high glucose‐mediated oxidative stress. Phytomedicine, 66, 153107. 31790903 10.1016/j.phymed.2019.153107

[eph13297-bib-0082] Tao, H. , Ge, G. , Liang, X. , Zhang, W. , Sun, H. , Li, M. , & Geng, D. (2020). ROS signaling cascades: Dual regulations for osteoclast and osteoblast. Acta Biochim Biophys Sin (Shanghai), 52(10), 1055–1062. 33085739 10.1093/abbs/gmaa098

[eph13297-bib-0083] Tao, Z.‐S. , Zhou, W.‐S. , Yang, M. , & Xu, H. (2020). Resveratrol reverses the negative effect of alcohol on hydroxyapatite‐coated implant osseointegration in senile female rats. Zeitschrift für Gerontologie und Geriatrie, 53(6), 538–545. 31435788 10.1007/s00391-019-01595-3

[eph13297-bib-0084] Terheyden, H. , Lang, N. P. , Bierbaum, S. , & Stadlinger, B. (2012). Osseointegration–communication of cells. Clinical Oral Implants Research, 23(10), 1127–1135. 22092345 10.1111/j.1600-0501.2011.02327.x

[eph13297-bib-0085] Ungvari, Z. , Sonntag, W. E. , de Cabo, R. , Baur, J. A. , & Csiszar, A. J. E. (2011). Mitochondrial protection by resveratrol. Exercise and Sport Sciences Reviews, 39(3), 128. 21383627 10.1097/JES.0b013e3182141f80 PMC3123408

[eph13297-bib-0086] Vaidya, P. , Mahale, S. , Kale, S. , & Patil, A. (2017). Osseointegration – A review. Journal of Medical and Dental Sciences, 16(01), 45–48.

[eph13297-bib-0087] Van den Bossche, J. , Baardman, J. , Otto, N. A. , van der Velden, S. , Neele, A. E. , van den Berg, S. M. , Luque‐Martin, R. , Chen, H. J. , Boshuizen, M. C. S. , Ahmed, M. , Hoeksema, M. A. , de Vos, A. F. , & de Winther, M. P. J. (2016). Mitochondrial dysfunction prevents repolarization of inflammatory macrophages. Cell Reports, 17(3), 684–696. 27732846 10.1016/j.celrep.2016.09.008

[eph13297-bib-0088] Wan, M. C. , Tang, X. Y. , Li, J. , Gao, P. , Wang, F. , Shen, M. J. , Gu, J. T. , Tay, F. , Chen, J. H. , Niu, L. N. , Xiao, Y. H. , & Jiao, K. (2021). Upregulation of mitochondrial dynamics is responsible for osteogenic differentiation of mesenchymal stem cells cultured on self‐mineralized collagen membranes. Acta Biomaterialia, 136, 137–146. 34571268 10.1016/j.actbio.2021.09.039

[eph13297-bib-0089] Wang, H. , Fu, X. , Shi, J. , Li, L. , Sun, J. , Zhang, X. , Han, Q. , Deng, Y. , & Gan, X. (2021). Nutrient element decorated polyetheretherketone implants steer mitochondrial dynamics for boosted diabetic osseointegration. Advanced Science (Weinheim), 8(20), 2101778. 10.1002/advs.202101778 PMC8529468 34396715

[eph13297-bib-0090] Wang, S. , Yang, J. , Lin, T. , Huang, S. , Ma, J. , & Xu, X. (2020). Excessive production of mitochondrion‑derived reactive oxygen species induced by titanium ions leads to autophagic cell death of osteoblasts via the SIRT3/SOD2 pathway. Molecular Medicine Reports, 22(1), 257–264. 32468046 10.3892/mmr.2020.11094 PMC7248520

[eph13297-bib-0091] Wang, X. , Li, Y. , Feng, Y. , Cheng, H. , & Li, D. (2020). The role of macrophages in osseointegration of dental implants: An experimental study in vivo. Journal of Biomedical Materials Research. Part A, 108(11), 2206–2216. 32363723 10.1002/jbm.a.36978

[eph13297-bib-0092] Wang, X. , Yu, Y. , Ji, L. , Geng, Z. , Wang, J. , & Liu, C. (2021). Calcium phosphate‐based materials regulate osteoclast‐mediated osseointegration. Bioactive Materials, 6(12), 4517–4530. 34632163 10.1016/j.bioactmat.2021.05.003 PMC8484898

[eph13297-bib-0093] Wang, Y. , Li, N. , Zhang, X. , & Horng, T. (2021). Mitochondrial metabolism regulates macrophage biology. Journal of Biological Chemistry, 297(1), 100904. 34157289 10.1016/j.jbc.2021.100904 PMC8294576

[eph13297-bib-0094] Wang, Y. , Zang, Q. S. , Liu, Z. , Wu, Q. , Maass, D. , Dulan, G. , Shaul, P. W. , Melito, L. , Frantz, D. E. , Kilgore, J. A. , Williams, N. S. , Terada, L. S. , & Nwariaku, F. E. (2011). Regulation of VEGF‐induced endothelial cell migration by mitochondrial reactive oxygen species. American Journal of Physiology. Cell Physiology, 301(3), C695–C704. 21653897 10.1152/ajpcell.00322.2010 PMC3174570

[eph13297-bib-0095] Wang, Y. , Zhang, Y. , & Miron, R. J. (2016). Health, maintenance, and recovery of soft tissues around implants. Clinical Implant Dentistry and Related Research, 18(3), 618–634. 25873299 10.1111/cid.12343

[eph13297-bib-0096] Wright, G. L. , Maroulakou, I. G. , Eldridge, J. , Liby, T. L. , Sridharan, V. , Tsichlis, P. N. , & Muise‐Helmericks, R. C. (2008). VEGF stimulation of mitochondrial biogenesis: Requirement of AKT3 kinase. The FASEB Journal, 22(9), 3264–3275. 18524868 10.1096/fj.08-106468 PMC2518259

[eph13297-bib-0097] Yang, D. , & Wan, Y. (2019). Molecular determinants for the polarization of macrophage and osteoclast. Seminars in Immunopathology, 41(5), 551–563. 31506868 10.1007/s00281-019-00754-3 PMC6815265

[eph13297-bib-0098] Yang, F. , Tang, J. , Dai, K. , & Huang, Y. (2019). Metallic wear debris collected from patients induces apoptosis in rat primary osteoblasts via reactive oxygen species‑mediated mitochondrial dysfunction and endoplasmic reticulum stress. Molecular Medicine Reports, 19(3), 1629–1637. 30628694 10.3892/mmr.2019.9825 PMC6390047

[eph13297-bib-0099] Yao, Y. , Cai, X. , Ren, F. , Ye, Y. , Wang, F. , Zheng, C. , Qian, Y. , & Zhang, M. (2021). The macrophage‐osteoclast axis in osteoimmunity and osteo‐related diseases. Frontiers in Immunology, 12, 1066. 10.3389/fimmu.2021.664871 PMC8044404 33868316

[eph13297-bib-0100] Yu, W. P. , Ding, J. L. , Liu, X. L. , Zhu, G. D. , Lin, F. , Xu, J. J. , Wang, Z. , & Zhou, J. L. (2021). Titanium dioxide nanotubes promote M2 polarization by inhibiting macrophage glycolysis and ultimately accelerate endothelialization. Immunity, Inflammation and Disease, 9(3), 746–757. 33835721 10.1002/iid3.429 PMC8342206

[eph13297-bib-0101] Yuan, Y. , Chen, Y. , Peng, T. , Li, L. , Zhu, W. , Liu, F. , Liu, S. , An, X. , Luo, R. , Cheng, J. , Liu, J. , & Lu, Y. (2019). Mitochondrial ROS‐induced lysosomal dysfunction impairs autophagic flux and contributes to M1 macrophage polarization in a diabetic condition. Clinical Science (London, England: 1979), 133(15), 1759–1777. 31383716 10.1042/CS20190672

[eph13297-bib-0102] Zhang, D. X. , & Gutterman, D. D. (2007). Mitochondrial reactive oxygen species‐mediated signaling in endothelial cells. American Journal of Physiology. Heart and Circulatory Physiology, 292(5), H2023–H2031. 17237240 10.1152/ajpheart.01283.2006

[eph13297-bib-0103] Zhang, L. , Haddouti, E.‐M. , Welle, K. , Burger, C. , Kabir, K. , & Schildberg, F. A. (2020). Local cellular responses to metallic and ceramic nanoparticles from orthopedic joint arthroplasty implants. International Journal of Nanomedicine, 15, 6705. 32982228 10.2147/IJN.S248848 PMC7494401

[eph13297-bib-0104] Zhang, Q. , Raoof, M. , Chen, Y. , Sumi, Y. , Sursal, T. , Junger, W. , Brohi, K. , Itagaki, K. , & Hauser, C. J. (2010). Circulating mitochondrial DAMPs cause inflammatory responses to injury. Nature, 464(7285), 104–107. 20203610 10.1038/nature08780 PMC2843437

[eph13297-bib-0105] Zhang, X. , Jiang, Y. , Mao, J. , Ren, X. , Ji, Y. , Mao, Y. , Chen, Y. , Sun, X. , Pan, Y. , Ma, J. , & Huang, S. (2021). Hydroxytyrosol prevents periodontitis‐induced bone loss by regulating mitochondrial function and mitogen‐activated protein kinase signaling of bone cells. Free Radical Biology and Medicine, 176, 298–311. 34610362 10.1016/j.freeradbiomed.2021.09.027

[eph13297-bib-0106] Zhang, Y. , Rohatgi, N. , Veis, D. J. , Schilling, J. , Teitelbaum, S. L. , & Zou, W. (2018). PGC1β organizes the osteoclast cytoskeleton by mitochondrial biogenesis and activation. Journal of Bone and Mineral Research, 33(6), 1114–1125. 29521005 10.1002/jbmr.3398 PMC6002881

[eph13297-bib-0107] Zhang, Y. , Zhu, X. , Wang, G. , Chen, L. , Yang, H. , He, F. , & Lin, J. (2020). Melatonin rescues the ti particle‐impaired osteogenic potential of bone marrow mesenchymal stem cells via the SIRT1/SOD2 signaling pathway. Calcified Tissue International, 107(5), 474–488. 32767062 10.1007/s00223-020-00741-z

[eph13297-bib-0108] Zheng, C.‐X. , Sui, B.‐D. , Qiu, X.‐Y. , Hu, C.‐H. , & Jin, Y. (2020). Mitochondrial regulation of stem cells in bone homeostasis. Trends in Molecular Medicine, 26(1), 89–104. 31126872 10.1016/j.molmed.2019.04.008

[eph13297-bib-0109] Zheng, Y. , Zhou, H. , Dunstan, C. R. , Sutherland, R. L. , & Seibel, M. J. (2013). The role of the bone microenvironment in skeletal metastasis. Journal of Bone Oncology, 2(1), 47–57. 26909265 10.1016/j.jbo.2012.11.002 PMC4723345

[eph13297-bib-0110] Zhou, A. , Yu, H. , Liu, J. , Zheng, J. , Jia, Y. , Wu, B. , & Xiang, L. (2020). Role of Hippo‐YAP signaling in osseointegration by regulating osteogenesis, angiogenesis, and osteoimmunology. Frontiers in Cell and Developmental Biology, 8, 780. 32974339 10.3389/fcell.2020.00780 PMC7466665

[eph13297-bib-0111] Zhu, F. , Wang, J. , Ni, Y. , Yin, W. , Hou, Q. , Zhang, Y. , Yan, S. , & Quan, R. (2021). Curculigoside protects against titanium particle‐induced osteolysis through the enhancement of osteoblast differentiation and reduction of osteoclast formation. Journal of Immunology Research, 2021, 1. 10.1155/2021/5707242 PMC8275416 34285923

[eph13297-bib-0112] Zorov, D. B. , Juhaszova, M. , & Sollott, S. J. (2014). Mitochondrial reactive oxygen species (ROS) and ROS‐induced ROS release. Physiological Reviews, 94(3), 909–950. 24987008 10.1152/physrev.00026.2013 PMC4101632

[eph13297-bib-0113] Zou, D. , Zhu, S. , Zhou, J. , He, J. , Wang, Y. , Xie, Z. , Han, W. , You, S. , & Huang, Y. (2011). Hypoxia‐inducible factor‐1 [alpha]: A potential factor for the enhancement of osseointegration between dental implants and tissue‐engineered bone. Dental Hypotheses, 2(3), 118.

